# The ability of the commercial inertial measurement unit-based gait system in determining Parkinson’s disease: A systematic review with meta-analysis of cross-sectional studies

**DOI:** 10.1371/journal.pone.0360793

**Published:** 2026-10-15

**Authors:** Jiahao Pan, Hui Tang

**Affiliations:** 1 Biomechanics & Neurorehabilitation Lab, Department of Physical Education, Xinjiang Normal University, Urumqi, Xinjiang, China; 2 Department of Kinesiology and Health Education, The University of Texas at Austin, Austin, Texas, United States of America; St Mary’s University, UNITED STATES OF AMERICA

## Abstract

The purpose of this study was to use the meta-analytic approach to investigate the ability of Inertial Measurement Unit-based commercial system measured gait variability and asymmetry in distinguishing individuals with Parkinson’s disease (PD) from healthy controls (HC). Cross-sectional studies included using commercial IMU-based systems measuring gait variability or asymmetry at any stage of PD patients and healthy adults were included. Studies were determined by searching for four electronic databases and cross-referencing. Dual selection and data abstraction of studies were conducted. Hedges’ g standardized mean difference was calculated for each variable and pooled using inverse heterogeneity mode. Statistically significant was determined by two tailed z-alpha values ≤0.05 and non-overlapping 95% CI. Heterogeneity was estimated using Q and I^2^ with alpha values ≤0.10 for Q considered statistically significant. Small-study effects were examined using doi plots and LFK index. A total of 1,725 studies were screened. Eventually, 9 studies representing up to 571 PD patients and 470 HC met the inclusion criteria. Overall gait variability was significantly greater in individuals with PD than in HC (Hedges’ g = 0.368, 95% CI 0.139–0.597). The multi-IMU subgroup also showed significantly greater gait variability (Hedges’ g = 0.397, 95% CI 0.221–0.574), whereas the one-IMU subgroup did not. However, the between-subgroup difference was not statistically significant, and the multi-IMU prediction interval included null. The primary analysis detected no statistically significant overall difference in gait asymmetry (Hedges’ g = 0.180, 95% CI −0.012–0.373). These findings provide preliminary evidence of an average group-level difference in gait variability but hard to establish individual-level discrimination, clinical utility, or the superiority of multi-IMU systems.

## Introduction

Parkinson’s disease (PD) is the second most common neurodegenerative disorder around the world [1]. Moreover, PD has shown the fastest increase in prevalence and disability among neurological disorders, establishing it as one of leading causes of disability globally [2]. Based on Global Burden of Disease 2021, the prevalence of PD was 149 cases per 100,000 [3], aligning with a recent meta-analysis of 151 cases per 100,000 [4]. Projections show a 112% increase in global PD cases from 2021 to 2050, reaching 25.2 million cases in 2050, based on a probabilistic Bayesian model averaging approach [3]. Especially, China will have nearly half of the PD patients in the world in 2030 [5].

As the increased prevalence, PD’s economic burden strains healthcare systems globally [6,7]. In the United States, the total cost of PD exceeds $51.9 billion, including $25.4 billion of direct medical costs and $26.5 billion of indirect and non-medical costs ($14.2 billion for patient and caregiver burden, $7.5 billion for non-medical costs, and $4.8 billion for disability income) [6]. These costs will rise to $79 billion by 2037 [6]. In China, direct medical costs per hospitalized PD patient (mean stay: 9.9 ± 8.6 days) reached $1,759.9 ± 4,787.7 in Hebei Province from 2019 to 2021 [7]. These examples demonstrate that PD inflicts escalating financial damage on societies worldwide.

Regular monitoring the degree of motor symptoms in PD patients is critical to reduce costs and improve patient outcomes. Monitoring supports management decisions, such as adherence to the exercise interventions or other treatment plans, eventually prolong their independent life and decrease the indirect cost [8,9]. Clinical rating scales (e.g., Unified Parkinson’s Disease Rating Scale Part III & Hoehn and Yahr scale) in PD are internationally accepted clinical tools to assess motor symptoms, severity, progression, and treatment efficiency [10–12]. However, their outcomes vary with clinicians’ skills and experience [13,14] and these assessments are time-consuming [15]. The variability and time demand limit daily use, risking suboptimal decision making. Thus, it is critical importance in development of objective and effective clinical diagnostic tools to monitor PD symptoms.

Given PD patients with gait abnormalities, measurements such as gait variability and asymmetry have been widely applied and have been demonstrated as the effective biomarkers [16,17]. This evaluation really relies on biomechanical instruments, such as motion capture system or pressure sensitive walkway [18,19]. Although these instruments produce quantified, reliable, and accurate gait measurements, limitations must be admitted including expensive, long set-up time, and lack of flexibility in the testing environment [19,20]. Inertial Measurement Units (IMUs) have promises as a solution for the current dilemma. This is because IMUs are accessible and sensitive digital sensors that can continuously collect kinematic data [21,22]. Assessing gait measurements from IMU data has indicated its clinical application for determining early-stage of PD or monitoring motor progression in PD [21,23,24]. However, studies vary considerably in the number and placement of IMUs (e.g., wrist, trunk, waist, shank) [25,26].

Ideally, a clinically scalable approach would use as few IMUs as possible and no placement worries. This goal is particularly relevant to smartphone-based assessment because smartphones contain embedded IMU and may serve as remote assessment platforms [21,22,27]. Moreover, such an approach could reduce dependence on specialized equipment and enable assessment in real-life environment [27]. A systematic review indicated the validity of a single IMU for gait measurements analysis and their ability to assist in clinical laboratories [28]. However, a single IMU primarily captures motion at its carrying location and may provide fewer segment-specific measurements than a multi-IMU configuration [28,29]. Therefore, it is necessary to initially summarize these laboratory studies and understand whether significant validation is present in distinguishing PD from healthy individuals using gait variability and asymmetry based on different numbers or placements of IMU.

This meta-analysis evaluates the ability of IMU-based commercial system measured gait variability and asymmetry in distinguishing individuals with PD from healthy controls (HC). We also investigated the subgroup effects from different numbers (1 vs. > 1) or body positions (lower back vs. other positions) in distinguishing individuals with PD from HC.

## Methods

### Overview

This systematic review with meta-analysis adhere to 2020 Preferred Reporting Items for Systematic Reviews and Meta-Analyses (PRISMA) guidelines (See S1 File in S1 Data) [30,31]. This protocol was pre-registered on the Open Science Framework at https://osf.io/.

### Eligibility criteria

Studies that met the following criteria were included: 1) cross-sectional studies, 2) at least two distinct groups compared: healthy adults and any stage of PD patients, 3) patients without physical impairment and neurodegenerative comorbidities, 4) gait variability or asymmetry available from each study using commercial IMU-based system during walk at their preferred speed, and 5) full-length peer-reviewed studies published in any language, assuming an English-language abstract was available, from January 1, 2012 to April 26, 2025. We The criteria for diagnosis of PD was conducted according to the Movement Disorder Society (MDS) Clinical Diagnostic Criteria [32] or United Kingdom Parkinson’s Disease Brain Bank Criteria [33] or neurologists/specialists from the Department of Neurology. Studies from our own independent searching were limited to published articles with small-study effects (publication bias, etc.) examined when limited to published articles in peer-reviewed journals. Studies from non-English languages sources were translated into English.

### Information sources

Four electronic databases were searched to identify eligible studies from January 2012 forward. Databases included: 1) PubMed, 2) Web of Science, 3) Scopus, and 4) IEEE, covering studies in any language with English abstracts.

### Search strategy

The search strategy was developed by both authors (JP and HT) using text words as well as medical subject heading (MeSH) associated with the ability of required measurements via IMU to comparison between PD and HC. The keywords included “Parkinson”, “inertial”, “IMU”, “accelerometer”, “gyroscope”, “magnetometer”, “smartphone”, “smartwatch”, and “control”. With oversite from the second author (HT), the first author (JP) conducted all electronic database searches. A copy of an example search query using PubMed can be found in S2 File in S1 Data. This PubMed search strategy was adapted for other database searches.

### Study selection

The first author (JP) imported all studies for screening into EndNote (V19.0, New York, USA) and eliminated duplicates both electronically and manually. Following PRISMA 2020 guidelines, the first (JP) and second (HT) authors independently screened all studies. For titles and abstracts that met inclusion criteria or presented uncertainty, the authors retrieved the full text of each study. When multiple reports of the same study existed, the authors selected the study providing the most comprehensive data relevant to the systematic review with meta-analysis. Neither author was blinded to the journal titles, study authors or institutions from where the work was derived. Reasons for exclusion were coded according to the PECOS criteria: (1) inappropriate population (P), (2) inappropriate exposure (E), inappropriate comparison (C), inappropriate outcome (O), inappropriate study design (S). No disagreements occurred regarding final study eligibility. Disagreements were limited to assigning the primary PECOS criteria for exclusion to some studies. These disagreements were resolved through an online discussion until both authors agreed on a single reason for exclusion.

### Data collection process

The first (JP) and second (HT) authors developed a codebook in Microsoft Excel (Microsoft, Washington, USA) prior to data abstraction. The major categories of variables included: 1) study characteristics (author, country, journal, year, one year impact force, sources of funding, etc.), 2) participant characteristics (age, height, body mass, gender, PD stages, medication condition etc.), 3) IMU characteristics (brand, number, location, etc.), 4) task protocol, and 5) outcome characteristics for gait variability and asymmetry (sample sizes, means, standard deviations, etc.). Following PRISMA 2020 guidelines, both authors independently abstracted data from all studies after completing the codebook. If there is 100% disagreement with a specific item, that item was examined across all studies. No discrepancies were identified between the independently extracted data.

### Primary outcomes

The primary outcome in this study was gait variability and asymmetry via IMU from different number and body positions. If the study included more than one measure that was categorized as gait variability or asymmetry (e.g., stride variability, step variability, stride time asymmetry, swing time asymmetry, etc.) or is from different subgroups, we pooled these results into a single outcome using decomposition of means and standard deviation.

### Risk of bias assessment in individual studies

In accordance with PRISMA 2020 guidelines, both first (JH) and second (HT) authors independently assessed risk of bias using the Joanna Briggs Institute (JBI) Critical Appraisal Tools [34]. The instrument is specific to analytical cross-sectional studies [34], which includes eight questions: 1) Were the criteria for inclusion in the sample clearly defined? 2) Were the study subjects and the setting described in detail? 3) Was the exposure measured in a valid and reliable way? 4) Were objective, standard criteria used for measurement of the condition? 5) Were confounding factors identified? 6) Were strategies to deal with confounding factors stated? 7) Were the outcomes measured in a valid and reliable way? and 8) Was appropriate statistical analysis used? Each question is scored for adequacy as “yes”, “no”, “unclear”, or “not applicable”. No study was excluded based on the results of the risk of bias assessment. Disagreements were resolved by consensus. The only disagreement concerned the checklist item 8 of Meng et al.’s study [35]. This disagreement was resolved through discussion during an online meeting, after which both authors agreed on the final rating.

### Data synthesis

Effect sizes were computed as standardized mean differences (SMD) using Hedges’ g to quantify group differences in IMU-measured gait metrics. Hedges’ g was calculated by subtracting control group, dividing the difference by the pooled standard deviation, and applying a correction for small-sample bias [36]. Thus, positive values indicated greater gait variability or asymmetry in individuals with PD than in HC. Studies reporting multiple PD subgroups were analyzed separately before pooling to yield one SMD per study. If standard deviations were unavailable, they were imputed from 95% confidence intervals or pre/post values using Follmann et al.’s methods [37].

We pooled effect size for gait variability or asymmetry using the inverse heterogeneity (IVhet) mode [38]. Two-sided *p* values <0.05 as well as non-overlapping 95% confidence intervals (CIs) that did not include zero were considered statistically significant. Additionally, we calculated 95% prediction intervals (PIs), which provide an estimate of what result one might expect if they conducted their own study as well as providing a better estimate of heterogeneity and inconsistency than the Q and *I*^*2*^ statistics because it is based on an absolute measure (tau) in its estimation [39]. Outlier analysis was conducted by deleting the results from those studies in which their 95% CIs did not fall within the pooled 95% CI. In addition, we examined subgroup differences between gait variability or asymmetry from IMU used from different numbers (1 vs. > 1) or body positions (lower back vs. other positions) in distinguishing individuals with PD from HC. Differences between subgroups were considered statistically significant using the between-subgroup Q test, with *p* < 0.05 considered statistically significant.

The Q [40] and *I*^*2*^ [41] statistics were still used to determine heterogeneity and inconsistency for gait variability or asymmetry score differences. An α level of ≤ 0.10 for Q is considered to represent statistically significant heterogeneity. Inconsistency based on *I*^*2*^*,* a relative measure, was categorized as very low (< 25%), low (25% to <50%), moderate (50% to <75%) or large (≥75%) [41].

We assessed small-study effects (publication bias, etc.) using the Doi plot, a normal quantile plot [42]. In addition, the LFK index was used as a quantitative measure for small-study effects [42]. The closer the LFK index value is to zero, the more symmetrical is the Doi plot [43]. Following established guidelines, LFK results were categorized as no asymmetry (LFK index within ±1), minor asymmetry (LFK index exceeds ±1 but within ±2), and major asymmetry (LFK index exceeds ±2) [42].

Because some studies reported multiple gait variability or asymmetry metrics measured in the same participants, we conducted sensitivity analyses to examine the influence of dependence among these metrics [44,45]. Each component metric was first converted to Hedges’ g. Metric-level effect estimates were then combined within each study using inverse-variance weighting while assuming common pairwise correlations (ρ) of 0, 0.25, 0.50, 0.75, and 0.90 [44]. For each assumed correlation, the covariance between two metric-level effects was calculated as ρ multiplied by the square root of the product of their sampling variances. The resulting study-level estimates were pooled using the IVhet model. Because the included studies did not report the complete covariance matrices needed to estimate these correlations directly, the selected values were treated as sensitivity assumptions rather than observed correlations.

### Statistical analysis

All statistical analyses were conducted using Stata SE (version 16.1), the user-written routine ‘admetan’ in Stata as well as the user-written routine ‘lfk’ in Stata. The first author (JP) initially conducted all statistical analyses. Metric-level correlation sensitivity analyses were implemented using study-level covariance matrices and inverse-variance weighting. The second author (HT) then replicated all analyses. No discrepancies were identified between the initial and replicated statistical analyses.

## Results

### Search results

A flow diagram of the search process for potentially eligible studies is shown in Fig 1. From 1,725 retrieved studies, 1,262 duplicates were removed (1,176 electronically, 86 manually), leaving 463. Of these 463 studies, 321 studies (69.3%) were excluded based on the title and abstract for one main reason, leaving a total of 142 studies that underwent full-text review. Based on the PECOS criteria, the proportion of each reason for exclusion were as follows: (1) inappropriate population (36.8%), (2) inappropriate exposure (2.8%), inappropriate comparison (16.5%), inappropriate outcome (11.3%), inappropriate study design (23.1), and others (9.3%). Of the 143 that underwent full-text review, 134 were excluded (93.7%), 9 because they recruited inappropriate population, 73 because they used inappropriate outcome, 3 because they carried out inappropriate comparison, 12 because they presented inappropriate exposure, 32 because they had inappropriate study design, and 4 because they had other reasons. Thus, a total of 9 studies were included representing up to 571 PD patients and 470 HC met the criteria for inclusion in this systematic review and meta-analysis [35,46–53]. Studies were published in 9 different journals [35,46–53] with one year impact factors ranging from 1.8 to 5.22 ($\overset{―}{X}$ ± SD = 3.6 ± 1.2, Mdn = 3.3) for the 8 studies (88.9%) in which data were available [46–53].

**Fig 1:**
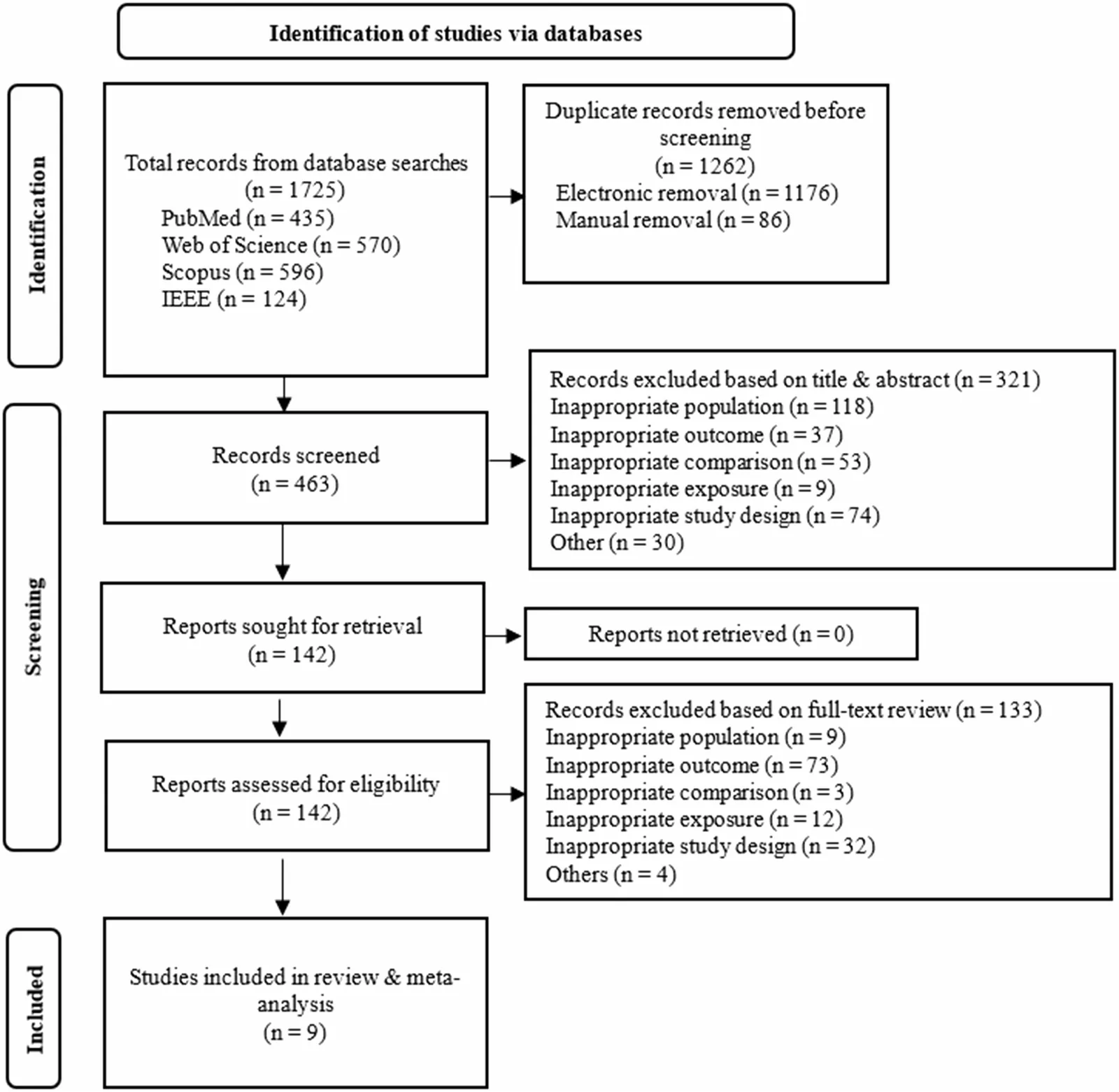
PRISMA flow diagram. Searches conduction into two phases.

### Study characteristics

Table 1 provided a list of study and participant characteristics ranging from year 2016–2025 [35,46–53]. These studies were conducted in 6 different countries; three in United Kingdom [46,49,50], two in United States [47,53], and one each in either Belgium [51], Netherlands [52], German [48], or China [35]. Six of 9 studies (66.7%) reported external funding for their research [48–53]; five from government sources [48–51,53], four from university sources [49,51–53], and three from other sources [48,50,52].

**Table 1:** Description of participants characteristics from each study that met the inclusion criteria.

Study and year	Country/Region	Groups	Age ($\overset{―}{X}\pm SD$)	M/F	MDS-UPDRS III ($\overset{―}{X}\pm SD)$	Medication	IMU (sample rate)	Location	Protocol	Outcomes
Del Din et al., 2016	United Kingdom	Stage 1–3 PD HC	66.9 ± 9.4 66.6 ± 7.7	20/10 15/15	29.5 ± 10.7	On	Axivity AX3 (50 Hz)	L5	Walk on a 25 meters track in their preferred speed for 2 mins	1. Step time variability 2. Stance time variability 3. Swing time variability 4. Step length variability 5. Step velocity variability 6. Step time asymmetry 7. Stance time asymmetry 8. Swing time asymmetry 9. Step length asymmetry
Gera et al., 2020	United States	Stage 1–3 PD HC	59.1 ± 6.8 62.7 ± 8.5	22/12 7/8	26.0 ± 18.0	On	APDM^TM^ Mobility Lab System (128 Hz)	L5 and dorsum of both feet	Walk 7 meters, turn 180 degrees, and return to the initial starting point at their preferred speed	1. Stride length asymmetry
Meng et al., 2023	China	Stage 1–2 PD HC	64.1 ± 7.0 63.4 ± 5.8	12/9 7/12	17.1 ± 10.7	On	Noraxon Mymotion^TM^ system (100 Hz)	Head, upper thoracic, pelvic, and both sides of upper arms, thighs, shanks, and feet	Five rounds of walking 5 meters, turn 180 degrees, and return to the initial starting point at their preferred speed	1. Stride length variability 2. Stride time variability 3. Stance time variability 4. Swing time variability 5. Stride length asymmetry 6. Stride time asymmetry 7. Stance time asymmetry 8. Swing time asymmetry
Micó-Amigo et al., 2025	Germany	prodromal PD HC	67.9 ± 4.3 68.0 ± 3.8	12/3 72/18	nd	nd	DynaPort^®^ Hybrid (100 Hz)	L5	Continuously walk 3 rounds around a cloth circle of a 1.2 m diameter at their preferred speed	1. Step time variability 2. Stride time variability 3. Step time asymmetry
Morris et al., 2023	United States	Stage 1–4 PD HC	68.0 ± 8.1 71.7 ± 8.0	210/124 70/74	24.1 ± 12.2	On	APDM^TM^ Mobility Lab System (128 Hz)	L5 and both feet	Walk on a 7 meters track at their preferred speed for 2 mins	1. Stride length variability 2. Stride time variability
Ur Rehman et al., 2019	United Kingdom	Stage 1–3 PD HC	69.2 ± 10.1 72.3 ± 6.7	59/34 49/54	32.4 ± 10.3	On	Axivity AX3 (100 Hz)	L5	Walk on a 25 meters track at their preferred speed for 2 mins	1. Step velocity variability 2. Step length variability 3. Step time variability 4. Stance time variability 5. Step time asymmetry 6. Swing time asymmetry 7. Stance time asymmetry
van Gelder et al., 2021	United Kingdom	Mild to Moderate PD HC	59.0 ± 7.0 50.0 ± 8.0	14/6 8/16	33.0 ± 9.0	nd	APDM^TM^ Mobility Lab System (128 Hz)	L5 and shin of both feet	Walk on a 10 meters track at their preferred speed for 6 mins	1. Gait asymmetry in the anterior-posterior direction 2. Gait asymmetry in the medial-lateral direction 3. Gait asymmetry in the superior-inferior direction
Warlop et al., 2017	Belgium	Stage 1–3 PD HC	62.2 ± 6.9 60.3 ± 4.8	9/5 3/7	27.5 ± 9.0	nd	Temec Instruments B.V. (512 Hz)	Lateral malleolus	Walk on a 42 meters track at their preferred speed for 12 mins	1. Stride time variability
Weersink et al., 2021	Netherlands	Stage 2–3 PD HC	65.0 ± 11.0 67.0 ± 9.0	17/10 17/18	nd	On	Compumedics Neuroscan (512 Hz)	L3 and both sides of Lateral malleolus	Walk through a 150 meters track from start to finish and back at their preferred speed	1. Stride time variability 2. Swing time asymmetry

$\overset{―}{X}\pm SD$, mean ± standard deviation; PD, Parkinson’s disease; HC, healthy control; MDS-UPDRS III, Movement Disorder Society-Sponsored Unified Parkinson’s Disease Rating Scale, Part III (Motor Examination); L5, fifth lumbar vertebra; and L3, third lumbar vertebra. Hoehn and Yahr Scale was used to define the 1–5 stage of PD. MDS-UPDRS was used to define the mild to severe of PD.

Six IMU-based commercial gait systems were included in these studies [35,46–53]; three using APDM^TM^ Mobility Lab System [47,50,53], two using Axivity AX3 [46,49], one each using either Noraxon Mymotion^TM^ system [35], DynaPort^®^ Hybrid [48], Temec Instruments B.V [51]., or Compumedics Neuroscan [52]. Four of 9 studies (44.4%) were attached to only one IMU sensor [46,48,49,51]; three on the fifth lumbar vertebra [46,48,49], and one on the lateral malleolus [51]. Five of 9 studies (55.6%) were attached to more than one IMU sensors [35,47,50,52,53]; all these studies included an IMU sensor on the lumbar vertebra [35,47,50,52,53]. Because there is not enough study comparison between gait variability or asymmetry from different body positions (lower back vs. other positions), so we did not conduct further analysis. All studies were conducted at their preferred walking speed including straight walking and turning [35,46–53], while 6 of 9 studies (66.7%) were reported both gait variability and asymmetry [35,46–49,52], 1 of 9 studies (11.1%) were only reported gait asymmetry [50], and 1 of 9 studies (11.1%) were only reported gait variability [51].

### Risk of bias assessment

Table 2 presented detailed information on risk of bias assessment using JBI Critical Appraisal Tools for cross-sectional studies. More than 88.9% of the studies were treated as mild to moderate risk of bias with respect to that: (1) 4 of 9 studies (44.4%) showed sampling weakness [46,49,50,52], (2) 7 of 9 studies (77.8%) presented confounding weakness [35,46–49,52,53], and (2) 1 of 9 studies (11.1%) has analysis weakness [35].

**Table 2:** Study-level risk of bias results using the Joanna Briggs Institute Critical Appraisal Tools.

Study and year	Q1	Q2	Q3	Q4	Q5	Q6	Q7	Q8
Del Din et al., 2016	0	1	1	1	–	–	1	1
Gera et al., 2020	1	1	1	1	–	–	1	1
Meng et al., 2023	1	1	1	1	–	–	1	0
Micó-Amigo et al., 2025	1	1	1	1	–	–	1	1
Morris et al., 2023	1	1	1	1	–	–	1	1
Ur Rehman et al., 2019	0	1	1	1	–	–	1	1
van Gelder et al., 2021	0	1	1	1	1	1	1	1
Warlop et al., 2017	1	1	1	1	1	1	1	1
Weersink et al., 2021	1	0	1	1	–	–	1	1

Q1: Were the criteria for inclusion in the sample clearly defined?

Q2: Were the study subjects and the setting described in detail?

Q3: Was the exposure measured in valid and reliable way?

Q4: Were objective, standard criteria used for measurement of the condition?

Q5: Were confounding factors identified?

Q6: Were strategies to deal with confounding factors stated?

Q7: Were the outcomes measured in a valid and reliable way?

Q8: Was appropriate statistical analysis used?

1, yes; 0, no; and -, unclear.

### Overall results for primary outcomes

#### Gait variability.

Overall results for difference in gait variability between PD and HC were presented in Table 3 and Fig 2. A total of 7 studies representing 958 participants were included (533 with PD and 425 HC) [35,46,48–53]. The pooled analysis showed significantly greater gait variability in individuals with PD than in HC (Hedges’ g = 0.368, 95% CI 0.139 to 0.597; z = 3.16, *p* = 0.002). Heterogeneity was not statistically significant (Q = 9.96, *p* = 0.126), and inconsistency was moderate (*I*² = 39.8%). Four studies had 95% CIs that included the null value [35,46,48,51]. At the individual study level, Hedges’ g ranged from −0.322 (95% CI −0.889 to 0.246) to 0.584 (95% CI −0.298 to 0.871). The 95% PI included null value (−0.157 to −0.893). The Doi plot for small-study effects indicated a major negative asymmetry (LFK index = −2.94), suggesting possible small-study effects (Fig 3). No study was identified as an outlier because every individual-study 95% CI overlapped the pooled 95% CI. Therefore, no outlier-excluded analysis was performed.

**Table 3:** Gait Variability and Asymmetrical Results. Parkinson’s Disease versus Healthy Controls.

Variable	ES (#)	N (#)	Hedges’ g (95% CI)	z (*p*)	Q (*p*)	*I^2^*	95% PI
Gait variability							
All	7	958	0.368 (0.139, 0.597)	3.16 (0.002) *	9.96 (0.126)	39.8%	-0.157, 0.893
-One IMU	4	378	0.322 (-0.183, 0.828)	1.25 (0.212)	9.18 (0.027)	67.3%	-1.593, 2.238
->One IMU	3	580	0.397 (0.221, 0.574)	4.42 (< 0.001) **	0.50 (0.777)	0.0%	-0.746, 1.540
Gait asymmetry							
All	7	549	0.180 (-0.012, 0.373)	1.84 (0.066) *	6.60 (0.360)	9.1%	-0.145, 0.505
-One IMU	3	354	0.201 (-0.273, 0.676)	0.83 (0.406)	5.71 (0.058)	65.0%	-4.777, 5.180
->One IMU	4	195	0.146 (-0.142, 0.434)	1.00 (0.319)	0.80 (0.849)	0.0%	-0.486, 0.779

Notes: ES(#), number of effects sizes; N(#), total number of participants; CI, confidence interval; Q, Cochran Q statistic for heterogeneity; PI, prediction interval; *, statistically significant (*p* < 0.05); **, statistically significant, *p* < 0.10.

**Fig 2:**
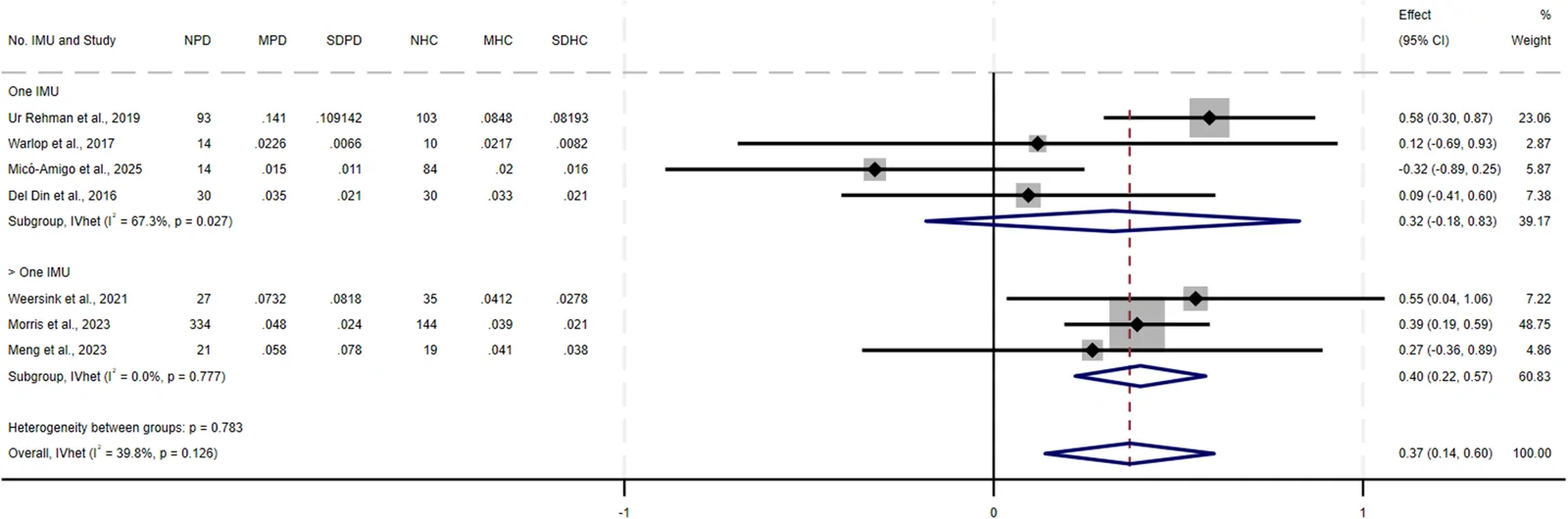
Forest plot for difference in gait variability between Parkinson’s patients and healthy control.

**Fig 3:**
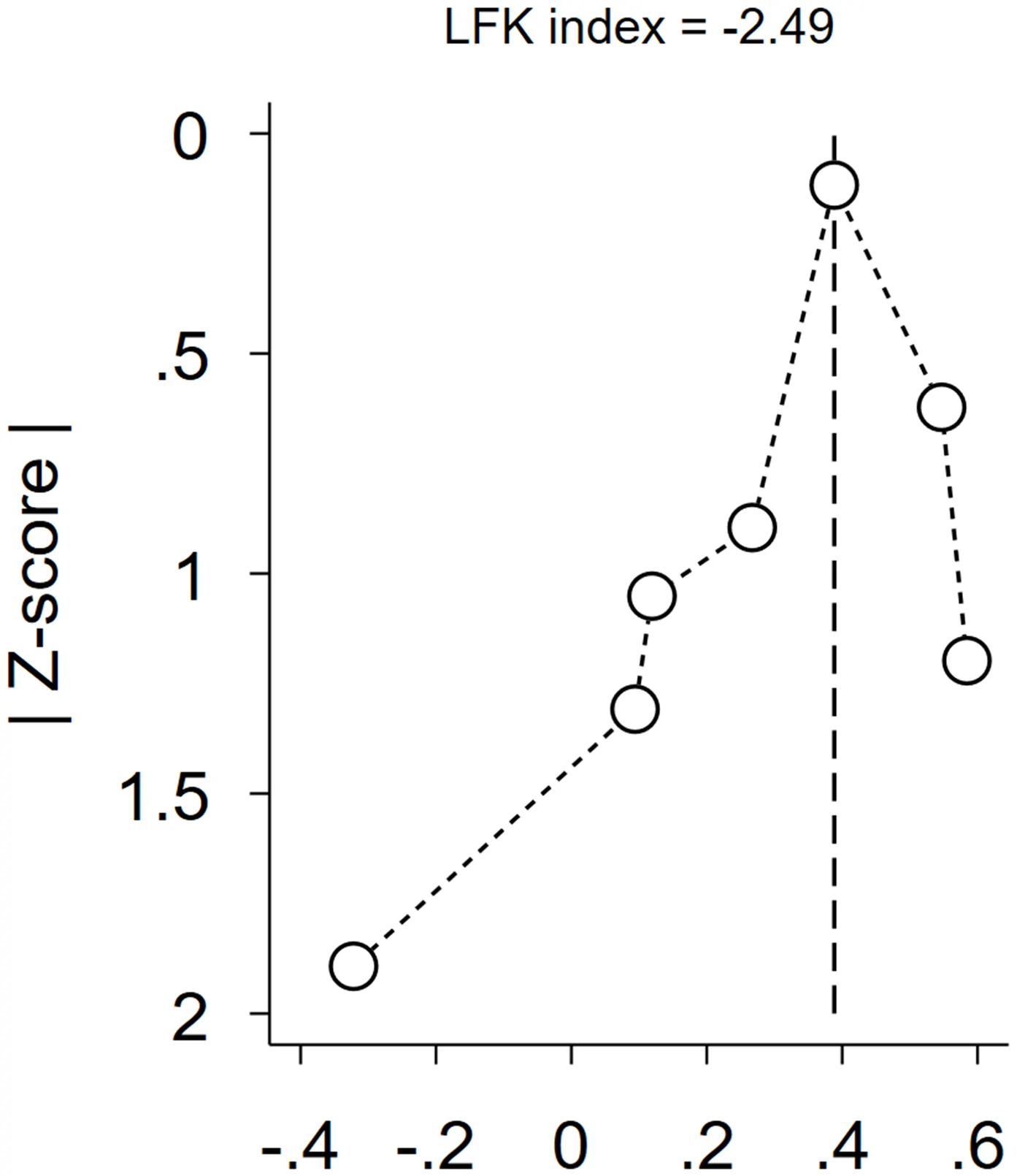
Doi plots for gait variability difference between Parkinson’s patients and healthy control that is generated via the Stata lfk package.

Results for subgroup analyses partitioned according to number of IMU sensors were shown in Table 3 and Fig 2. Studies using more than one IMU showed significantly greater gait variability in individuals with PD than in HC (Hedges’ g = 0.397, 95% CI 0.221 to 0.574; z = 4.42, *p* < 0.001). No significant difference in gait variability was observed between groups when using only one IMU sensor at lumbar vertebra or lateral malleolus (Hedges’ g = 0.322, 95% CI −0.183 to 0.828; z = 1.25, *p* = 0.212). Heterogeneity was observed among studies using one IMU (Q = 9.18, *p* = 0.027), with moderate inconsistency (*I*² = 67.3%). In contrast, heterogeneity was not statistically significant among studies using more than one IMU (Q = 0.50, *p* = 0.777), with very low inconsistency (*I*² = 0.0%). The 95% PI included the null for both the one-IMU subgroup (−1.593 to 2.238) and the multiple-IMU subgroup (−0.746 to 1.540). No statistically significant between-group differences were found (Q between = 0.08, *p* = 0.783).

In sensitivity analyses treating the component gait variability metrics as separate but correlated outcomes (see S3 File in S1 Data), the overall effect remained statistically significant across assumed within-study correlations ranging from 0 to 0.90 (Hedges’ g = 0.421 to 0.505; all *p* ≤ 0.001). The multi-IMU subgroup also remained statistically significant across all correlation assumptions (Hedges’ g = 0.346 to 0.396; all *p* < 0.001). In contrast, the one-IMU subgroup remained nonsignificant (Hedges’ g = 0.460 to 0.609; *p* = 0.061 to 0.083), and no significant difference was found between the one-IMU and multi-IMU subgroups (Q between = 0.06 to 0.55; p = 0.460 to 0.806). Thus, the overall and multi-IMU gait-variability findings were robust to the assumed correlations among metrics.

#### Gait asymmetry.

Overall results for difference in gait asymmetry index between PD and HC were presented in Table 3 and Fig 4. A total of 7 studies representing 549 participants were included (239 with PD and 310 HC) [35,46–50,52]. The pooled analysis showed no statistically significant difference in gait asymmetry between individuals with PD and HC (Hedges’ g = 0.180, 95% CI −0.012 to 0.373; z = 1.84, *p* = 0.066). Heterogeneity was not statistically significant (Q = 6.60, *p* = 0.360), and inconsistency was very low (*I*² = 9.1%). Six of the seven studies had 95% CIs that included the null value [35,46–48,50,52]. At the individual study level, Hedges’ g ranged from −0.425 (95% CI −0.995 to 0.144) to 0.348 (95% CI 0.066 to 0.631). The 95% PI included null value (−0.145 to 0.505). The Doi plot showed major negative asymmetry (LFK index = −2.03, Fig 5), indicating possible small-study effects. No study was identified as an outlier because every individual-study 95% CI overlapped the pooled 95% CI. Therefore, no outlier-excluded analysis was performed.

**Fig 4:**
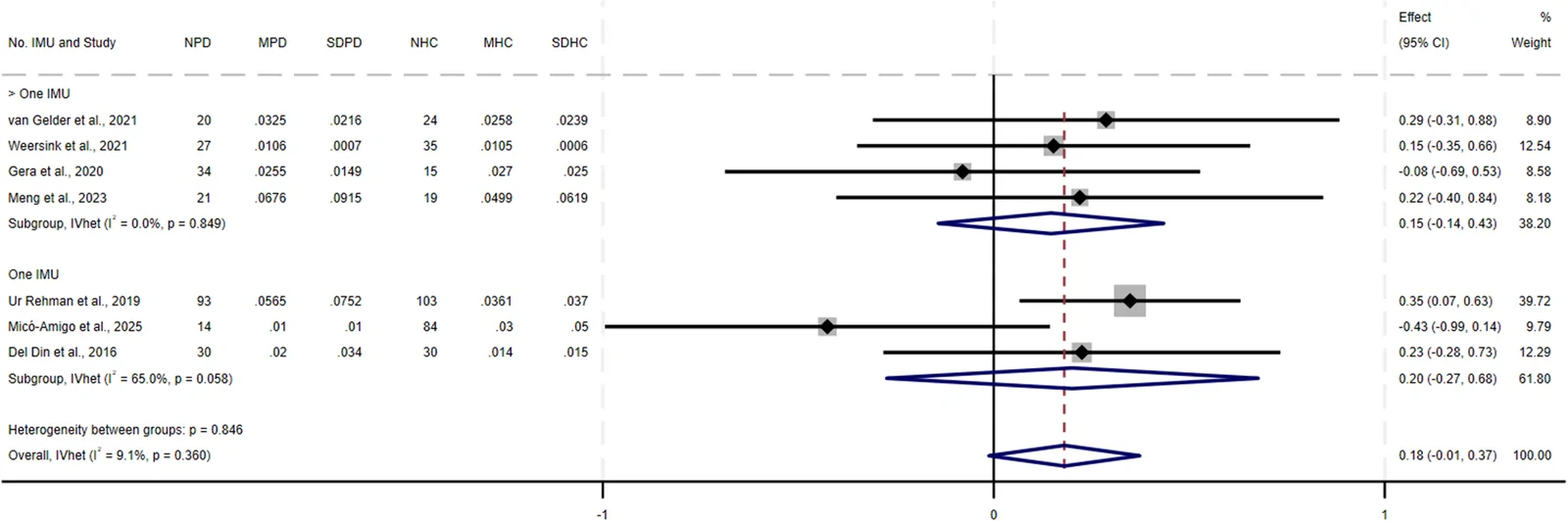
Forest plot for difference in gait asymmetry between Parkinson’s patients and healthy control.

**Fig 5:**
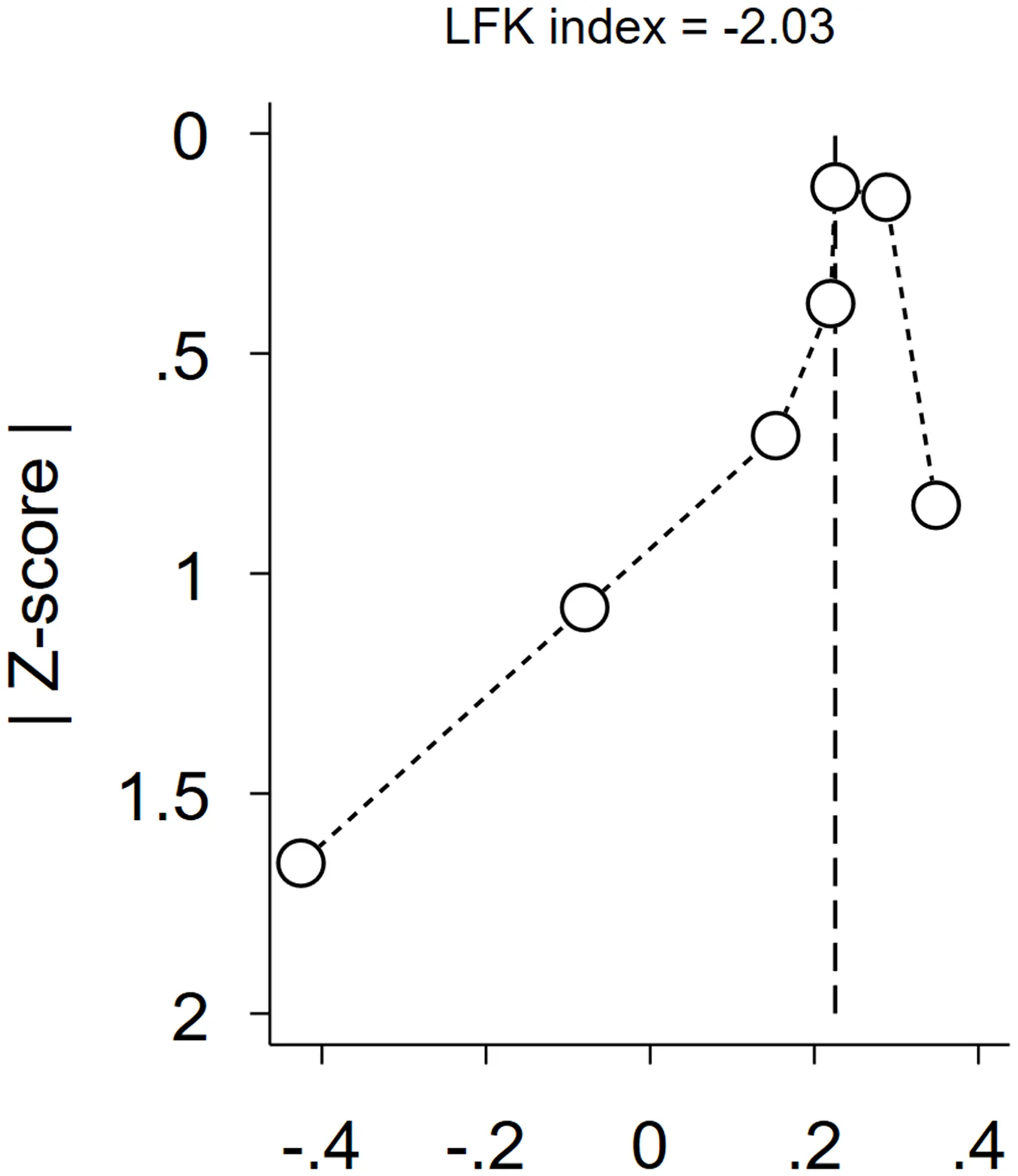
Doi plots for gait asymmetry difference between Parkinson’s patients and healthy control that is generated via the Stata lfk package.

Results for subgroup analyses partitioned according to number of IMU sensors were shown in Table 3 and Fig 4. Our study also did not observe significant difference in gait asymmetry when using either one IMU sensor (Hedges’ g = 0.201, 95% CI −0.273 to 0.676; z = 0.83, *p* = 0.406) or more than one IMU sensor (Hedges’ g = 0.146, 95% CI −0.142 to 0.434; z = 1.00, *p* = 0.319). Heterogeneity was observed using one IMU (Q = 5.71, *p* = 0.058), with moderate inconsistency (*I*² = 65.0%). Additionally, heterogeneity was not statistically significant among studies using more than one IMU (Q = 0.80, *p* = 0.849), with very low inconsistency (*I*² = 0.0%). The 95% PI included the null for both the one-IMU subgroup (−4.777 to 5.180) and the multiple-IMU subgroup (−0.486 to 0.779). No statistically significant between-group differences were found (Q between = 0.04, *p* = 0.846).

The gait asymmetry results were more sensitive to the treatment of multiple outcomes (see S3 File in S1 Data). When the component metrics were analyzed separately, the overall pooled effect was positive across all assumed correlations (Hedges’ g = 0.246 to 0.389; *p* = 0.003 to 0.050), although the result was borderline when a correlation of 0.90 was assumed. The multi-IMU subgroup was statistically significant when correlations of 0 and 0.25 were assumed (Hedges’ g = 0.333 and 0.259, respectively) but was not statistically significant under assumptions of 0.50 to 0.90 (Hedges’ g = 0.195 to 0.224; *p* = 0.102 to 0.183). The one-IMU subgroup and the between-subgroup comparison remained nonsignificant under all correlation assumptions. These results indicate that the gait asymmetry findings were sensitive to how dependence among multiple metrics was handled.

## Discussion

### Overall findings

The primary purpose of the current systematic review with meta-analysis was to investigate the ability of IMU-based gait variability and asymmetry to distinguish individuals with PD from HC. The pooled analysis showed significantly greater gait variability in individuals with PD, whereas no significant group difference was detected for gait asymmetry. However, the 95% prediction intervals for both outcomes included null value. Therefore, the findings provide preliminary evidence of an average group-level difference in gait variability but do not establish the ability of these outcomes to discriminate between PD and HC at the individual level.

We further investigated the subgroup effects from IMUs used in different numbers (1 vs. > 1) or body positions (lower back vs. other positions) in distinguishing individuals with PD from HC. A formal comparison of lower back versus other sensor placements was not possible because only one gait variability study did not include a lower back, lumbar, or pelvic sensor [51], and no corresponding comparator study was available for gait asymmetry. Moreover, several multi-IMU systems included sensors at both lower back and other body positions, preventing the effects of placement from being separated from sensor number and system configuration.

Studies using more than one IMU showed significantly greater gait variability in individuals with PD than in HC, whereas studies using one IMU showed no statistically significant difference. However, the difference between these subgroups was not statistically significant, and both prediction intervals included null value. Therefore, the findings do not demonstrate that increasing the number of IMUs improves diagnostic performance. The statistically significant pooled multi-IMU effect represents an average group-level difference and does not directly establish individual-level discrimination. Because only three studies contributed to this subgroup and its 95% prediction interval included the null, its clinical utility remains uncertain [35,52,53]. In the primary analysis, no significant gait asymmetry difference was observed in either IMU-number subgroup. Although the gait variability findings were robust across the assumed within-study correlations, the gait asymmetry findings varied according to the assumed dependence among metrics and should therefore be interpreted cautiously.

### Implications for research

The current findings suggest several areas to consider for future research on distinguishing individuals with PD from HC using IMU-based outcomes. First, since only seven studies included either gait variability or gait asymmetry to investigate the difference between PD and HC, future studies in investigating the ability of the commercial IMU-based gait outcomes in determining Parkinson’s disease appear warranted. Second, our included studies did not examine the difference among different stages of PD and HC. In practice, initiating treatment at clinical diagnosis stage (later than stage 2) may fail to prove optimal or effective due to the advanced neurodegenerative state when outstanding motor symptoms are present [54]. In other words, the pre-diagnostic phase of PD (before stage 2) offers a window of opportunity in which disease-modifying therapies could be most beneficial, especially those aimed at delaying or preventing the progression to overt disease and its many complications [54,55]. Thus, there is enthusiasm for early disease detection in PD patients to facilitate the development of disease-modifying therapies. Third, given that the studies used different outcomes related to gait variability (e.g., step time variability, stance time variability, swing time variability, step length variability, step velocity variability, etc.) or asymmetry (e.g., step time asymmetry, stance time asymmetry, swing time asymmetry, step length asymmetry, etc.), these outcomes were combined within studies before Hedges’ g was calculated. This approach did not explicitly account for correlations among multiple outcomes measured in the same participants and may have affected both the magnitude and precision of the pooled estimates. However, sensitivity analyses that treated the metrics separately showed that the gait variability findings were robust across assumed correlations, whereas the gait asymmetry findings varied according to these assumptions. Future studies should report individual gait parameters together with their covariance or correlation matrices, allowing multivariate analyses that appropriately account for within-study dependence. Additionally, sensor placement may provide another explanation for the absence of detectable gait asymmetry. Of the seven studies reporting asymmetry, six used sensors exclusively on the trunk or lower limbs. The remaining study included upper-arm sensors, but we did not consider arm swing asymmetry in this study. Consequently, the pooled analysis primarily represented lower limb asymmetry and could not directly capture reduced or asymmetric arm swing. This is clinically relevant because arm swing asymmetry may be detectable during early or prodromal PD, even when asymmetry in lower-limb spatiotemporal parameters remains subtle [56,57]. Future studies should include bilateral wrist or upper arm sensors and analyze upper- and lower-body asymmetry separately. Fourth, only one study did not use IMU sensor on participants’ lower back [51]. In fact, many previous studies have demonstrated that gait outcomes using the IMU attached on other body positions (e.g., wrist, mid-thigh, mid/lateral aspect of shank, lateral/medial aspect of malleolus, dorsum of foot, and midsole) also presented acceptable accuracy [29,51,58]. Moreover, gait outcomes from IMU embedded into midsole could present higher precision than IMU attached on lower back [29]. Future studies should also focus on examining the ability of IMU-based gait outcomes from different body positions to distinguish individuals at various stages of PD from HC. Such information is critical for the development of reliable diagnostic tools, as integrating these multidimensional gait features into advanced AI models could enhance classification accuracy, improve robustness across diverse populations, and enable early detection of subtle gait abnormal. Fifth, all included studies evaluated gait variability and asymmetry only during walking protocols. Within the subgroups using more than one IMU, gait variability was significantly greater in individuals with PD than in HC. Clinically, increased gait variability represents less consistent stride to stride regulation of temporal or spatial gait parameters [59,60]. Greater gait variability has been reported in individuals with freezing of gait, even during walking without an overt freezing episode [59]. This association suggests that gait variability may reflect an underlying susceptibility to freezing [59]. It has also been associated with postural instability and a greater risk of falling [60]. However, the included studies did not consistently classify gait phenotypes, we cannot determine which disturbance contributed most to the observed variability. Future studies should distinguish these phenotypes and examine whether combining gait variability with tremor, bradykinesia, rigidity, and postural instability improves PD classification [1]. These multimodal data could also be evaluated using appropriately developed and externally validated AI models. While we must admit that only three studies contributed to this subgroup, its 95% prediction interval included the null, and the difference between the one-IMU and multi-IMU subgroups was not statistically significant [35,52,53]. This finding therefore represents preliminary evidence of an average group-level difference rather than evidence of individual-level discrimination or superior clinical utility for multi-IMU systems. Sixth, since more than half of the studies were considered to be at high or unclear risk of bias with respect to confounding issues, future studies should address these issues in their experimental design and report such information. Finally, the results of this study appear to have limited implications for practice. Future evidence syntheses should examine other IMU-derived motor outcomes across standing, walking, turning, and manual tasks when sufficiently comparable outcomes and clinically relevant reference standards are available.

### Strengths and potential limitations

A major strength of this study is that it was the first, to our knowledge, to investigate the ability of IMU-based commercial systems measured gait variability and asymmetry in distinguishing individuals with PD from HC. This is important given the prevalence of PD [3,4], its expected future increase [3,5], and its associated financial burden [6,7]. Specifically, the portability and scalability of IMU-based systems may facilitate large-scale, repeated collection of gait data across clinical and real-world settings. Such data could improve the characterization of gait impairment and its progression across diverse PD populations [21,23,24]. When applied within representative cohorts and validated against clinical diagnoses, AI-based analysis of large-scale IMU data may contribute to population screening and complement conventional estimates of PD prevalence and disease burden in the future [21,27]. Compared with laboratory-based motion capture systems and pressure sensitive walkways, commercial IMU systems are portable, may require less specialized infrastructure, and can facilitate assessment outside laboratory environments [19–22,27]. Systems using fewer sensors, particularly smartphones with embedded IMUs, may further reduce equipment and setup requirements and support repeated or remote assessment [21,22,27]. Collectively, these characteristics suggest that IMU-based systems may provide a resource efficient and potentially cost-effective platform for supporting clinical assessment, longitudinal monitoring, and rehabilitation management in individuals with PD. Second, a novel approach, the IVhet model, was used to pool findings [38]. This resulted in more robust estimates as well as a lower observed variance than those derived from the traditional random effects model, regardless of heterogeneity [38]. This is noteworthy given the need to provide the most accurate results possible when examining the ability of selected outcome(s) from IMU in distinguishing individuals with PD from HC. Third, the Doi plot was used to assess small-study effects (publication bias, etc.) [42]. This resulted in more intuitive illustration and robust estimates small-study effects than the often-used funnel plot [42].

Several potential limitations should be acknowledged. First, the sample sizes for meta- analyses were small for our subgroups. Thus, the strength of these associations is weak. Second, given the small sample sizes and missing data expected, we did not conduct any type of meta-regression analyses leading to undiscovering the potential sources of bias. Third, estimating the gait variability and asymmetry may not have accurately reflected the true number of those in each group from each study. To conduct meta-analysis, we simply pooled those results from different outcomes related to gait variability or asymmetry. Fourth, similar to any data meta-analysis of cross-sectional studies, the temporal relationship between exposure and outcome cannot be established, limiting the ability to draw causal inferences. Fifth, sensor placement could not be examined independently because nearly all studies included a lower-back, lumbar, or pelvic sensor. Multi-IMU systems also frequently combined these sensors with other placements. Consequently, placement was confounded with sensor number and system configuration, and an adequate comparator subgroup was unavailable. Sixth, cross-sectional studies are more prone to heterogeneity, residual confounding, and selection bias, which may influence the strength and direction of the pooled associations. The findings should be interpreted as descriptive of associations rather than indicative of causality. Seventh, the included studies compared individuals with PD only with healthy controls and did not include individuals with other parkinsonian disorders, such as multiple system atrophy or progressive supranuclear palsy. Therefore, our findings cannot establish whether IMU-derived gait variability and asymmetry can differentiate PD from other clinically similar parkinsonian disorders. Future studies should directly compare these individuals to determine the clinical utility of IMU-derived gait outcomes for differential diagnosis. Finally, the results in the current meta-analysis could not be generalizable to early-stage PD.

## Conclusions

Gait variability measured by using more than one IMU was significantly greater in individuals with PD than in HC. However, the small number of studies, the null-inclusive prediction interval, and the nonsignificant between-subgroup comparison preclude conclusions about diagnostic utility. Therefore, a need exists for additional, well-designed, cross-sectional trails on this topic.

## Supporting information

S1 Data S1 File. PRISMA 2020 checklist. S2 File. Search query. An example of search query from PubMed. S3 File. Sensitivity analyses of pooled gait variability and gait asymmetry effects. Sensitivity analyses of pooled gait variability and gait asymmetry effects under alternative assumed within-study correlations among metric-level effect estimates.
(ZIP)
