# Supplementary material for: The ability of the commercial inertial measurement unit-based gait system in determining Parkinson’s disease: A systematic review with meta-analysis of cross-sectional studies
Source: PLoS One. 2026 Oct 15;21(10):e0360793. doi: 10.1371/journal.pone.0360793 (PMC13671562; doi:10.1371/journal.pone.0360793)
Supplement: Supplementary file 1 [file pone.0360793.s001.zip › S2 File.docx]

**Search query.**
