# Supplementary material for: The ability of the commercial inertial measurement unit-based gait system in determining Parkinson’s disease: A systematic review with meta-analysis of cross-sectional studies
Source: PLoS One. 2026 Oct 15;21(10):e0360793. doi: 10.1371/journal.pone.0360793 (PMC13671562; doi:10.1371/journal.pone.0360793)
Supplement: Supplementary file 1 [file pone.0360793.s001.zip › S3 File.docx]

S3 Table. Sensitivity analyses of pooled gait variability and gait asymmetry effects under alternative assumed within-study correlations among metric-level effect estimates.

| Assumed ρ | Overall variability, *g* (95% CI) | Multi-IMU variability, *g* (95% CI) | Overall asymmetry, *g* (95% CI) | Multi-IMU asymmetry, *g* (95% CI) |
| --- | --- | --- | --- | --- |
| 0.00 | 0.505 (0.197-0.812), *p* = 0.001 | 0.346 (0.244-0.448), *p* < 0.001 | 0.389 (0.132-0.647), *p* = 0.003 | 0.333 (0.147-0.519), *p* < 0.001 |
| 0.25 | 0.454 (0.194-0.714), *p* = 0.001 | 0.381 (0.244-0.518), *p* < 0.001 | 0.330 (0.071-0.590), *p* = 0.013 | 0.259 (0.014-0.503), *p* = 0.038 |
| 0.50 | 0.435 (0.193-0.677), *p* < 0.001 | 0.389 (0.236-0.542), *p* < 0.001 | 0.291 (0.038-0.545), *p* = 0.024 | 0.224 (-0.045-0.492), *p* = 0.102 |
| 0.75 | 0.425 (0.199-0.651), *p* < 0.001 | 0.394 (0.229-0.559), *p* < 0.001 | 0.261 (0.013-0.510), *p* = 0.039 | 0.203 (-0.078-0.484), *p* = 0.156 |
| 0.90 | 0.421 (0.205-0.637), *p* < 0.001 | 0.396 (0.224-0.568), *p* < 0.001 | 0.246 (0.000-0.493), *p* = 0.050 | 0.195 (-0.092-0.481), *p* = 0.183 |

Note: ρ, assumed common pairwise within-study correlation; CI, confidence interval.
