# Supplementary material for: The ability of the commercial inertial measurement unit-based gait system in determining Parkinson’s disease: A systematic review with meta-analysis of cross-sectional studies
Source: PLoS One. 2026 Oct 15;21(10):e0360793. doi: 10.1371/journal.pone.0360793 (PMC13671562; doi:10.1371/journal.pone.0360793)
Supplement: Supplementary file 2 [file pone.0360793.s003.docx]

Thank you for submitting your manuscript to PLOS ONE. After careful consideration, we feel that it has merit but does not fully meet PLOS ONE’s publication criteria as it currently stands. Therefore, we invite you to submit a revised version of the manuscript that addresses the points raised during the review process.

Response:

We would like to appreciate the opportunity to revise and resubmit our manuscript. We have seriously considered all the comments and carefully revised the manuscript accordingly. Revisions are highlighted in the manuscript using a red color to indicate where changes have taken place. We feel that the quality of the manuscript has been significantly improved with these modifications and improvements based on the reviewers’ suggestions and comments.

Journal Requirements:

When submitting your revision, we need you to address these additional requirements.

1. Please ensure that your manuscript meets PLOS ONE's style requirements, including those for file naming. The PLOS ONE style templates can be found at

https://journals.plos.org/plosone/s/file?id=wjVg/PLOSOne_formatting_sample_main_body.pdf and

<https://journals.plos.org/plosone/s/file?id=ba62/PLOSOne_formatting_sample_title_authors_affiliations.pdf>

Response:

The format has been revised based on PLOS ONE's style requirements.

2. Thank you for stating the following in your Competing Interests section:

“No authors have competing interests”

Please complete your Competing Interests on the online submission form to state any Competing Interests. If you have no competing interests, please state "The authors have declared that no competing interests exist.", as detailed online in our guide for authors at http://journals.plos.org/plosone/s/submit-now

This information should be included in your cover letter; we will change the online submission form on your behalf.

Response:

It has been revised in our main text and added in our cover letter (underlined).

3. When completing the data availability statement of the submission form, you indicated that you will make your data available on acceptance. We strongly recommend all authors decide on a data sharing plan before acceptance, as the process can be lengthy and hold up publication timelines. Please note that, though access restrictions are acceptable now, your entire data will need to be made freely accessible if your manuscript is accepted for publication. This policy applies to all data except where public deposition would breach compliance with the protocol approved by your research ethics board. If you are unable to adhere to our open data policy, please kindly revise your statement to explain your reasoning and we will seek the editor's input on an exemption. Please be assured that, once you have provided your new statement, the assessment of your exemption will not hold up the peer review process.

Response:

We added the Data Availability Statement in our main text. We shared all our data in the Figshare: https://doi.org/10.6084/m9.figshare.33140492.

4. Please include captions for your Supporting Information files at the end of your manuscript, and update any in-text citations to match accordingly. Please see our Supporting Information guidelines for more information: <http://journals.plos.org/plosone/s/supporting-information>.

Response:

It has been added.

5. If the reviewer comments include a recommendation to cite specific previously published works, please review and evaluate these publications to determine whether they are relevant and should be cited. There is no requirement to cite these works unless the editor has indicated otherwise.

Response:

Understood that.

Reviewer #1: Thank you for the opportunity to review this interesting manuscript examining the ability of IMU-based gait systems to distinguish individuals with Parkinson’s disease (PD) from healthy controls. The topic is clinically relevant, and the systematic approach taken by the authors is valuable. I would like to offer several comments that may help strengthen the manuscript.

Response:

We appreciate the reviewer’s positive assessment and constructive suggestions. The comments helped us improve the alignment of the Introduction with the study objective, strengthen the clinical interpretation of the results, clarify the study’s scope and practical implications, and make the consensus procedures more transparent.

1.Consistency between the Introduction and the study objective

The Introduction emphasizes the importance of monitoring the progression of motor symptoms in PD and the associated economic burden. However, the primary aim of the present study is to evaluate the ability of IMU-based gait measures to distinguish individuals with PD from healthy controls. To improve the overall coherence of the manuscript, the Introduction may benefit from placing greater emphasis on the clinical significance of early detection and differential identification between PD and healthy individuals, rather than disease progression.

Response:

We thank the reviewer for identifying this mismatch. We agree that the original Introduction placed disproportionate emphasis on disease progression and economic burden relative to the review question. We have refocused the Introduction on the clinical need for timely, objective identification of subtle gait abnormalities and on the potential complementary role of IMU-derived measures in distinguishing PD from healthy controls based on different numbers and placements of IMU. These revisions appear in the Introduction as follows:

Given PD patients with gait abnormalities, measurements such as gait variability and asymmetry have been widely applied and have been demonstrated as the effective biomarkers [16, 17]. This evaluation really relies on biomechanical instruments, such as motion capture system or pressure sensitive walkway [18, 19]. Although these instruments produce quantified, reliable, and accurate gait measurements, limitations must be admitted including expensive, long set-up time, and lack of flexibility in the testing environment [19, 20]. Inertial Measurement Units (IMUs) have promises as a solution for the current dilemma. This is because IMUs are accessible and sensitive digital sensors that can continuously collect kinematic data [21, 22]. Assessing gait measurements from IMU data has indicated its clinical application for determining early-stage of PD or monitoring motor progression in PD [21, 23, 24]. However, studies vary considerably in the number and placement of IMUs (e.g., wrist, trunk, waist, shank) [25, 26].

Ideally, a clinically scalable approach would use as few IMUs as possible and no placement worries. This goal is particularly relevant to smartphone-based assessment because smartphones contain embedded IMU and may serve as remote assessment platforms [21, 22, 27]. Moreover, such an approach could reduce dependence on specialized equipment and enable assessment in real-life environment [27]. A systematic review indicated the validity of a single IMU for gait measurements analysis and their ability to assist in clinical laboratories [28]. However, a single IMU primarily captures motion at its carrying location and may provide fewer segment-specific measurements than a multi-IMU configuration [28, 29]. Therefore, it is necessary to initially summarize these laboratory studies and understand whether significant validation is present in distinguishing PD from healthy individuals using gait variability and asymmetry based on different numbers or placements of IMU.

New references:

18.	Godinho C, Domingos J, Cunha G, Santos AT, Fernandes RM, Abreu D, et al. A systematic review of the characteristics and validity of monitoring technologies to assess Parkinson’s disease. J Neuroeng Rehabil. 2016;13(1):24. doi: https://doi.org/10.1186/s12984-016-0136-7.

19.	Jakob V, Küderle A, Kluge F, Klucken J, Eskofier BM, Winkler J, et al. Validation of a Sensor-Based Gait Analysis System with a Gold-Standard Motion Capture System in Patients with Parkinson’s Disease. Sensors. 2021;21(22):7680. doi: https://doi.org/10.3390/s21227680.

20.	Chen P-H, Wang R-L, Liou D-J, Shaw J-S. Gait disorders in Parkinson's disease: assessment and management. Int J Gerontol. 2013;7(4):189-93. doi: https://doi.org/10.1016/j.ijge.2013.03.005.

22.	Brognara L, Palumbo P, Grimm B, Palmerini L. Assessing gait in Parkinson’s disease using wearable motion sensors: a systematic review. Diseases. 2019;7(1):18. doi: https://doi.org/10.3390/diseases7010018.

28.	Mobbs RJ, Perring J, Raj SM, Maharaj M, Yoong NKM, Sy LW, et al. Gait metrics analysis utilizing single-point inertial measurement units: A systematic review. Mhealth. 2022;8:9. doi: https://doi.org/10.21037/mhealth-21-17.

2.Clinical interpretation of gait variability

The manuscript reports that gait variability measured by more than one IMU sensor is associated with distinguishing PD from healthy controls. However, the clinical interpretation of “gait variability” remains somewhat unclear. From a clinical perspective, it would be helpful if the authors could discuss what aspects of PD gait this variability might reflect—for example, whether it is more closely related to freezing of gait, general shuffling gait, or other gait disturbances commonly observed in PD. Adding such discussion would enhance the clinical relevance of the findings.

Response:

We thank the reviewer for requesting a clearer clinical interpretation of gait variability. We have expanded the fifth point in the Implications for Research section to explain that gait variability represents inconsistent stride-to-stride regulation of temporal and spatial gait parameters rather than a specific gait disturbance. We now discuss evidence that greater gait variability may reflect susceptibility to freezing of gait, including during walking without an overt freezing episode, and may also be associated with postural instability and an increased risk of falling. We further clarify that gait variability is distinct from shuffling gait, which is primarily characterized by shortened steps and reduced foot clearance. Because the included studies did not consistently classify participants according to specific gait phenotypes, our pooled findings cannot determine whether the observed variability primarily reflected freezing, shuffling, postural instability, or another gait disturbance. We have acknowledged this limitation and recommended that future studies distinguish these phenotypes and examine them alongside other PD motor manifestations.

The following text has been added to the Implications for Research section:

Fifth, all included studies evaluated gait variability and asymmetry only during walking protocols. Within the subgroups using more than one IMU, gait variability was significantly greater in individuals with PD than in HC. Clinically, increased gait variability represents less consistent stride to stride regulation of temporal or spatial gait parameters [54, 55]. Greater gait variability has been reported in individuals with freezing of gait, even during walking without an overt freezing episode [54]. This association suggests that gait variability may reflect an underlying susceptibility to freezing [54]. It has also been associated with postural instability and a greater risk of falling [55]. However, the included studies did not consistently classify gait phenotypes, we cannot determine which disturbance contributed most to the observed variability. Future studies should distinguish these phenotypes and examine whether combining gait variability with tremor, bradykinesia, rigidity, and postural instability improves PD classification [1]. These multimodal data could also be evaluated using appropriately developed and externally validated AI models.

New references:

56.	Hausdorff J, Schaafsma J, Balash Y, Bartels A, Gurevich T, Giladi N. Impaired regulation of stride variability in Parkinson's disease subjects with freezing of gait. Exp Brain Res. 2003;149(2):187-94. doi: https://doi.org/10.1007/s00221-002-1354-8.

57.	Schaafsma JD, Giladi N, Balash Y, Bartels AL, Gurevich T, Hausdorff JM. Gait dynamics in Parkinson's disease: relationship to Parkinsonian features, falls and response to levodopa. J Neurol Sci. 2003;212(1-2):47-53. doi: https://doi.org/10.1016/S0022-510X(03)00104-7.

3.Lack of detectable gait asymmetry

Parkinson’s disease is typically characterized by motor asymmetry, particularly in the early stages. Therefore, the absence of a significant difference in gait asymmetry between PD and healthy controls may appear somewhat unexpected from a clinical standpoint. It would be helpful if the authors could discuss possible explanations for this finding. For instance, the placement of sensors (e.g., on the trunk or lower limbs) may limit the ability to detect certain asymmetric features such as reduced arm swing.

Response:

We thank the reviewer for this important observation. We agree that sensor placement may partly explain the absence of a significant difference in gait asymmetry between individuals with PD and HC. We have expanded the third point in the Implications for Research section to discuss two methodological explanations. First, the included studies reported several biomechanically distinct asymmetry measures, which we combined into a single outcome. This aggregation may have obscured measure-specific differences. Second, six of the seven studies reporting asymmetry used sensors exclusively on the trunk or lower limbs. Although the remaining study included upper-arm sensors, it did not report arm-swing asymmetry. Therefore, our pooled analysis primarily represented trunk and lower-limb asymmetry and could not directly capture reduced or asymmetric arm swing. We now clarify that arm-swing asymmetry may be evident during early or prodromal PD, even when lower-limb spatiotemporal asymmetry is subtle. Accordingly, the non-significant pooled result should not be interpreted as evidence that motor asymmetry is absent in PD. We also recommend that future studies use bilateral wrist or upper-arm sensors and analyze upper- and lower-body asymmetry separately.

The following text has been added:

Additionally, sensor placement may provide another explanation for the absence of detectable gait asymmetry. Of the seven studies reporting asymmetry, six used sensors exclusively on the trunk or lower limbs. The remaining study included upper-arm sensors, but we did not consider arm swing asymmetry in this study. Consequently, the pooled analysis primarily represented lower limb asymmetry and could not directly capture reduced or asymmetric arm swing. This is clinically relevant because arm swing asymmetry may be detectable during early or prodromal PD, even when asymmetry in lower-limb spatiotemporal parameters remains subtle [53, 54]. Future studies should include bilateral wrist or upper arm sensors and analyze upper- and lower-body asymmetry separately.

New references:

53.	Mirelman A, Bernad‐Elazari H, Thaler A, Giladi‐Yacobi E, Gurevich T, Gana‐Weisz M, et al. Arm swing as a potential new prodromal marker of Parkinson's disease. Mov Disord. 2016;31(10):1527-34. doi: https://doi.org/10.1002/mds.26720Digital.

54.	Koh S-B, Park Y-M, Kim M-J, Kim W-S. Influences of elbow, shoulder, trunk motion and temporospatial parameters on arm swing asymmetry of Parkinson's disease during walking. Hum Mov Sci. 2019;68:102527. doi: https://doi.org/10.1016/j.humov.2019.102527.

4.Consideration of cost-effectiveness

Since the Introduction discusses the economic burden of PD and presents IMU systems as a potentially low-cost solution, it would be useful to include some discussion of the practical cost-effectiveness of the system. For example, how does the cost or practicality of this approach compare with other commonly used clinical assessments or diagnostic methods? Even a brief discussion would strengthen the practical implications of the study.

Response:

We thank the reviewer for this important suggestion. We agree that the practical and economic implications of IMU-based assessment should be considered. We have expanded the Introduction to explain that, compared with laboratory-based motion capture systems and pressure sensitive walkways, IMU-based systems may require less specialized infrastructure and enable data collection in clinical, remote, and real-world settings. Systems using fewer sensors, particularly smartphones with embedded IMUs, may further reduce equipment and setup requirements. We have also expanded the Strengths and Potential Limitations section to discuss the potential application of IMU-based systems as a resource efficient platform for supporting clinical diagnosis, longitudinal monitoring, and rehabilitation management in individuals with PD. Collectively, these characteristics suggest that IMU-based systems may provide a resource efficient and potentially cost-effective platform.

The following text has been added to the Strengths and Potential Limitations section:

This is important given the prevalence of PD [3, 4], its expected future increase [3, 5], and its associated financial burden [6, 7]. Specifically, the portability and scalability of IMU-based systems may facilitate large-scale, repeated collection of gait data across clinical and real-world settings. Such data could improve the characterization of gait impairment and its progression across diverse PD populations [21, 23, 24]. When applied within representative cohorts and validated against clinical diagnoses, AI-based analysis of large-scale IMU data may contribute to population screening and complement conventional estimates of PD prevalence and disease burden in the future [21, 27]. Compared with laboratory-based motion capture systems and pressure sensitive walkways, commercial IMU systems are portable, may require less specialized infrastructure, and can facilitate assessment outside laboratory environments [19-22, 27]. Systems using fewer sensors, particularly smartphones with embedded IMUs, may further reduce equipment and setup requirements and support repeated or remote assessment [21, 22, 27]. Collectively, these characteristics suggest that IMU-based systems may provide a resource efficient and potentially cost-effective platform for supporting clinical assessment, longitudinal monitoring, and rehabilitation management in individuals with PD.

5.Other parkinsonian disorders

The present analysis focuses on distinguishing PD from healthy controls. However, in clinical practice, an important diagnostic challenge is the differentiation of PD from other parkinsonian disorders, such as multiple system atrophy (MSA) or progressive supranuclear palsy (PSP). Since these conditions were not considered in the analysis, it would be appropriate to acknowledge this point as a limitation of the study.
Response:

We thank the reviewer for highlighting this important limitation. We agree that differentiating PD from other Parkinsonian disorders represents an important clinical challenge. Because the studies included in our meta-analysis compared individuals with PD only with healthy controls, our findings cannot establish whether IMU-derived gait variability and asymmetry can differentiate PD from other Parkinsonian disorders. We have therefore acknowledged this issue in the Strengths and Potential Limitations section and recommended direct comparisons with other Parkinsonian disorders in future studies.

The following text has been added:

“Sixth, the included studies compared individuals with PD only with healthy controls and did not include individuals with other parkinsonian disorders, such as multiple system atrophy or progressive supranuclear palsy. Therefore, our findings cannot establish whether IMU-derived gait variability and asymmetry can differentiate PD from other clinically similar parkinsonian disorders. Future studies should directly compare these individuals to determine the clinical utility of IMU-derived gait outcomes for differential diagnosis.”

6.Transparency of the consensus process

In the Methods section, the manuscript states that “disagreements were resolved by consensus.” While this is common practice in systematic reviews, the description would benefit from slightly greater transparency. If possible, the authors could briefly clarify what types of disagreements occurred and how consensus was achieved.

Response:

We thank the reviewer for this helpful suggestion. We have expanded the Methods to describe the disagreements encountered at each stage and how they were resolved. During study selection, no disagreements occurred regarding final study eligibility. Disagreements were limited to assigning the primary PECOS-based reason for excluding some studies and were resolved through an online discussion until both reviewers agreed on a single reason for exclusion. No discrepancies were identified during independent data extraction. In the risk-of-bias assessment, the only disagreement concerned the rating assigned to Meng et al. [35] for JBI checklist item 8, “Was appropriate statistical analysis used?” This disagreement was resolved through an online discussion, after which both reviewers agreed on the final rating. Finally, no discrepancies were identified between the initial and independently replicated statistical analyses. These clarifications have been added to the Study selection, Data collection process, Risk of bias assessment, and Statistical analysis subsections of the Methods.

The new read is as follows:

Study selection:

No disagreements occurred regarding final study eligibility. Disagreements were limited to assigning the primary PECOS criteria for exclusion to some studies. These disagreements were resolved through an online discussion until both authors agreed on a single reason for exclusion.

Data collection process:

No discrepancies were identified between the independently extracted data.

Risk of bias assessment in individual studies:

The only disagreement concerned the checklist item 8 of Meng et al.’s study [35]. This disagreement was resolved through discussion during an online meeting, after which both authors agreed on the final rating.

Statistical analysis:

No discrepancies were identified between the initial and replicated statistical analyses.

Overall, this study addresses an important topic, and with some clarification and expansion of the discussion, the manuscript could provide valuable insights into the potential role of IMU-based gait analysis in PD research.

Response:

We thank the reviewer again for these suggestions, which have improved the focus, clinical interpretation, and methodological transparency of the manuscript.
