# Supplementary material for: The ability of the commercial inertial measurement unit-based gait system in determining Parkinson’s disease: A systematic review with meta-analysis of cross-sectional studies
Source: PLoS One. 2026 Oct 15;21(10):e0360793. doi: 10.1371/journal.pone.0360793 (PMC13671562; doi:10.1371/journal.pone.0360793)
Supplement: Supplementary file 3 [file pone.0360793.s004.docx]

We thank reviewers’ feedback and comments.

Reviewer #2: *Critique*

This is a timely and well-structured PRISMA-guided meta-analysis on a clinically relevant question. Strengths include dual independent screening/extraction, use of the IVhet model, Doi plots/LFK for small-study effects, clear subgrouping by number of sensors, and transparent reporting of heterogeneity and prediction intervals. The main finding—that multi-IMU gait variability (but not asymmetry or single-IMU measures) shows a statistically significant difference—is cautious and appropriately tempered by the small number of studies and residual confounding risk.

Key limitations that weaken the strength of the conclusions:

- Very small evidence base (only 7–9 studies; subgroups even smaller), so the “practically important” claim for multi-IMU variability rests on limited data and is vulnerable to single-study influence.
- Substantial clinical and methodological heterogeneity (different commercial systems, sensor placements, walking protocols, PD stages, medication status, and pooling of multiple variability/asymmetry metrics via decomposition) is only partially addressed; the non-overlapping CIs criterion for subgroup differences is conservative but under-powered.
- Risk-of-bias assessment correctly flags frequent confounding and sampling issues, yet these are not quantitatively explored (no meta-regression).
- Pooling heterogeneous spatiotemporal metrics into single “variability” or “asymmetry” outcomes risks under-estimating true effects and obscures which specific parameters drive discrimination.
- Generalizability to early/prodromal PD and differential diagnosis versus other parkinsonisms remains untested, limiting translational impact.

Overall, the paper is methodologically sound for its scope but over-reaches slightly in language about practical importance given the sparse, heterogeneous data.

*Questions*
1. Why was the primary analysis performed on mean differences rather than standardized mean differences throughout, and how sensitive are the multi-IMU findings to the choice of effect-size metric?

**Response:**

Thank you for identifying this important methodological issue. We agree that mean differences were not the most appropriate effect-size metric because the included studies assessed gait variability and asymmetry using different spatiotemporal parameters and measurement scales. In the original analysis, we combined multiple measurements within each study before pooling the resulting mean differences. However, those estimates remained dependent on the units and scales of the component outcomes. We acknowledge that this was a limitation of the original analysis.

We have therefore replaced the mean-difference analyses throughout the manuscript with standardized mean differences corrected for small-sample bias using Hedges’ g. Positive values indicate greater gait variability or asymmetry in individuals with PD than in HC. We recalculated all study-level and pooled effects, confidence intervals, prediction intervals, heterogeneity statistics, subgroup analyses, between-subgroup tests, outlier assessments, and Doi plots. Table 3, Figs 2-5, the Results, Discussion, Abstract, and Conclusion have been updated accordingly. Because no study met the prespecified outlier criterion, the previous outlier-excluded figures and analyses were removed.

**The revised Data synthesis in method section states:**

“Effect sizes were computed as standardized mean differences (SMD) using Hedges’ g to quantify group differences in IMU-measured gait metrics. Hedges’ g was calculated by subtracting control group, dividing the difference by the pooled standard deviation, and applying a correction for small-sample bias [36]. Thus, positive values indicated greater gait variability or asymmetry in individuals with PD than in HC. Studies reporting multiple PD subgroups were analyzed separately before pooling to yield one SMD per study. If standard deviations were unavailable, they were imputed from 95% confidence intervals or pre/post values using Follmann et al.'s methods [37].”

“We pooled effect size for gait variability or asymmetry using the inverse heterogeneity (IVhet) mode [38]. Two-sided *p* values <0.05 as well as non-overlapping 95% confidence intervals (CIs) that did not include zero were considered statistically significant. Additionally, we calculated 95% prediction intervals (PIs), which provide an estimate of what result one might expect if they conducted their own study as well as providing a better estimate of heterogeneity and inconsistency than the Q and *I^2^* statistics because it is based on an absolute measure (tau) in its estimation [39]. Outlier analysis was conducted by deleting the results from those studies in which their 95% CIs did not fall within the pooled 95% CI. In addition, we examined subgroup differences between gait variability or asymmetry from IMU used from different numbers (1 vs. > 1) or body positions (lower back vs. other positions) in distinguishing individuals with PD from HC. Differences between subgroups were considered statistically significant using the between-subgroup Q test, with *p* <0.05 considered statistically significant.”

New reference:

Hedges LV. Distribution theory for Glass's estimator of effect size and related estimators. J Educ Stat. 1981;6(2):107-28. doi: https://doi.org/10.3102/10769986006002107.

**Sensitivity of the multi-IMU findings to the effect-size metric**

The principal multi-IMU conclusions were qualitatively unchanged after replacing mean differences with Hedges’ g.

For gait variability, the original mean-difference analysis showed significantly greater variability in individuals with PD when more than one IMU was used (weighted mean difference = 0.009, 95% CI 0.003 to 0.016; *p* = 0.004). The standardized analysis produced the same direction and statistical conclusion (Hedges’ g = 0.397, 95% CI 0.22 1to 0.574; *p* <0.001). Thus, the average multi-IMU gait-variability finding was not sensitive to the choice between these two effect-size metric.

For gait asymmetry, the original analysis did not detect a statistically significant multi-IMU effect (z = 0.62, *p* = 0.538). This conclusion was unchanged after standardization (Hedges’ g = 0.146, 95% CI 0.142 to 0.434; *p* = 0.319). Therefore, the primary analysis did not identify a detectable multi-IMU gait-asymmetry effect under either effect-size specification.

The overall gait-variability conclusion was sensitive to the selected effect-size metric. The original mean-difference analysis did not detect a significant overall group difference, whereas the analysis using Hedges’ g showed significantly greater gait variability in individuals with PD (Hedges’ g = 0.368, 95% CI 0.139 to 0.597; *p* = 0.002). We have reported this change transparently and revised the Results and Discussion accordingly.

The between-subgroup tests remained nonsignificant. For gait variability, the difference between studies using one IMU and those using more than one IMU was not statistically significant (Q_between = 0.08, *p* = 0.783). The corresponding subgroup difference for gait asymmetry was also nonsignificant (Q_between = 0.04, *p* = 0.846). Therefore, the analyses do not demonstrate that systems using more than one IMU produce significantly larger group differences than systems using one IMU.

**The revised Gait variability Results state:**

“Overall results for difference in gait variability between PD and HC were presented in Table 3 and Fig 2. A total of 7 studies representing 958 participants were included (533 with PD and 425 HC) [35, 44, 46-51]. The pooled analysis showed significantly greater gait variability in individuals with PD than in HC (Hedges’ g = 0.368, 95% CI 0.139 to 0.597; z = 3.16, *p* = 0.002). Heterogeneity was not statistically significant (Q = 9.96, *p* = 0.126), and inconsistency was moderate (*I*² = 39.8%). Four studies had 95% CIs that included the null value [35, 44, 46, 49]. At the individual study level, Hedges’ g ranged from -0.322 (95% CI -0.889 to 0.246) to 0.584 (95% CI -0.298 to 0.871). The 95% PI included null value (-0.157 to -0.893). The Doi plot for small-study effects indicated a major negative asymmetry (LFK index = -2.94), suggesting possible small-study effects (Fig 3). No study was identified as an outlier because every individual-study 95% CI overlapped the pooled 95% CI. Therefore, no outlier-excluded analysis was performed.

Results for subgroup analyses partitioned according to number of IMU sensors were shown in Table 3 and Fig 2. Studies using more than one IMU showed significantly greater gait variability in individuals with PD than in HC (Hedges’ g = 0.397, 95% CI 0.221 to 0.574; z = 4.42, *p* < 0.001). No significant difference in gait variability was observed between groups when using only one IMU sensor at lumbar vertebra or lateral malleolus (Hedges’ g = 0.322, 95% CI -0.183 to 0.828; z = 1.25, *p* = 0.212). Heterogeneity was observed among studies using one IMU (Q = 9.18, *p* = 0.027), with moderate inconsistency (*I*² = 67.3%). In contrast, heterogeneity was not statistically significant among studies using more than one IMU (Q = 0.50, *p* = 0.777), with very low inconsistency (*I*² = 0.0%). The 95% PI included the null for both the one-IMU subgroup (-1.593 to 2.238) and the multiple-IMU subgroup (-0.746 to 1.540). No statistically significant between-group differences were found (Q between = 0.08, *p* = 0.783).”

**The revised Gait asymmetry Results state:**

“Overall results for difference in gait asymmetry index between PD and HC were presented in Table 3 and Fig 4. A total of 7 studies representing 549 participants were included (239 with PD and 310 HC) [35, 44-48, 50]. The pooled analysis showed no statistically significant difference in gait asymmetry between individuals with PD and HC (Hedges’ g = 0.180, 95% CI -0.012 to 0.373; z = 1.84, *p* = 0.066). Heterogeneity was not statistically significant (Q = 6.60, *p* = 0.360), and inconsistency was very low (*I*² = 9.1%). Six of the seven studies had 95% CIs that included the null value [35, 44-46, 48, 50]. At the individual study level, Hedges’ g ranged from -0.425 (95% CI -0.995 to 0.144) to 0.348 (95% CI 0.066 to 0.631). The 95% PI included null value (-0.145 to 0.505). The Doi plot showed major negative asymmetry (LFK index = -2.03, Fig 5), indicating possible small-study effects. No study was identified as an outlier because every individual-study 95% CI overlapped the pooled 95% CI. Therefore, no outlier-excluded analysis was performed.

“Results for subgroup analyses partitioned according to number of IMU sensors were shown in Table 3 and Fig 4. Our study also did not observe significant difference in gait asymmetry when using either one IMU sensor (Hedges’ g = 0.201, 95% CI -0.273 to 0.676; z = 0.83, *p* = 0.406) or more than one IMU sensor (Hedges’ g = 0.146, 95% CI -0.142 to 0.434; z = 1.00, *p* = 0.319). Heterogeneity was observed using one IMU (Q = 5.71, *p* = 0.058), with moderate inconsistency (*I*² = 65.0%). Additionally, heterogeneity was not statistically significant among studies using more than one IMU (Q = 0.80, *p* = 0.849), with very low inconsistency (*I*² = 0.0%). The 95% PI included the null for both the one-IMU subgroup (-4.777 to 5.180) and the multiple-IMU subgroup (-0.486 to 0.779). No statistically significant between-group differences were found (Q between = 0.04, *p* = 0.846).”

Although the multi-IMU gait-variability effect remained statistically significant under both effect-size specifications, its interpretation remains limited. Only three studies contributed to this subgroup, its 95% prediction interval included zero (-0.746 to 1.540), and the difference between the one-IMU and multi-IMU subgroups was not statistically significant. Therefore, the reanalysis provides preliminary evidence of an average group-level difference but does not establish superior diagnostic performance or clinical utility for multi-IMU systems.

**The revised Discussion states:**

“Studies using more than one IMU showed significantly greater gait variability in individuals with PD than in HC, whereas studies using one IMU showed no statistically significant difference. However, the difference between these subgroups was not statistically significant, and both prediction intervals included null value. Therefore, the findings do not demonstrate that increasing the number of IMUs improves diagnostic performance. The statistically significant pooled multi-IMU effect represents an average group-level difference and does not directly establish individual-level discrimination. Because only three studies contributed to this subgroup and its 95% prediction interval included the null, its clinical utility remains uncertain [35, 52, 53]. In the primary analysis, no significant gait asymmetry difference was observed in either IMU-number subgroup. Although the gait variability findings were robust across the assumed within-study correlations, the gait asymmetry findings varied according to the assumed dependence among metrics and should therefore be interpreted cautiously.”

**The revised Conclusion states:**

“Gait variability measured by using more than one IMU was significantly greater in individuals with PD than in HC. However, the small number of studies, the null-inclusive prediction interval, and the nonsignificant between-subgroup comparison preclude conclusions about diagnostic utility. Therefore, a need exists for additional, well-designed, cross-sectional trails on this topic.”

2. Given that multiple variability (or asymmetry) outcomes were pooled within studies via decomposition of means/SDs, can you provide the within-study correlation assumptions used and any sensitivity analyses that treat the metrics as separate?

**Response:**

Thank you for raising this important issue. We agree that multiple gait variability or asymmetry metrics obtained from the same participants are statistically dependent and that this dependence was not explicitly incorporated when the component means and standard deviations were combined in the primary analysis. No within-study correlation assumption was used in that decomposition procedure.

To evaluate the influence of this dependence, we conducted additional sensitivity analyses in which the component metrics were treated separately. Each metric was first converted to Hedges’ g. Metric-level effect estimates were then combined within each study using inverse-variance weighting while assuming common pairwise correlations (ρ) of 0, 0.25, 0.50, 0.75, and 0.90. For each assumed correlation, the covariance between two metric-level effects was calculated as ρ multiplied by the square root of the product of their sampling variances. The resulting study-level estimates were pooled using the same IVhet model as the primary analysis. Because the included studies did not report the complete covariance matrices required to estimate these correlations directly, the selected correlation values were treated as sensitivity assumptions rather than observed correlations.

The gait variability findings were robust across the assumed correlations. The overall pooled effect remained statistically significant under all assumptions (Hedges’ g = 0.421 to 0.505; all *p* ≤ 0.001). The multi-IMU subgroup also remained statistically significant (Hedges’ g = 0.346 to 0.396; all *p* < 0.001), whereas the one-IMU subgroup remained nonsignificant (Hedges’ g = 0.460 to 0.609; *p* = 0.061to 0.083). The difference between the one-IMU and multi-IMU subgroups was nonsignificant under every assumed correlation (Q between = 0.06 to 0.55; *p* = 0.460 to 0.806). These findings support the robustness of the overall and multi-IMU gait-variability results to alternative assumptions regarding dependence among the component metrics.

The gait asymmetry findings were more sensitive to these assumptions. When the metrics were treated separately, the overall pooled effect was positive across the assumed correlations (Hedges’ g = 0.246 to 0.389; *p* = 0.003 to 0.050), although it was borderline when ρ = 0.90 was assumed. The multi-IMU subgroup was statistically significant when correlations of 0 and 0.25 were assumed but became nonsignificant under assumptions of 0.50 to 0.90 (Hedges’ g = 0.195 to 0.224; *p* = 0.102 to 0.183). The one-IMU subgroup and the between-subgroup comparison remained nonsignificant under all assumptions. We have therefore revised the manuscript to clarify that the gait asymmetry findings are sensitive to how dependence among multiple metrics is handled and should be interpreted cautiously.

We added the sensitivity-analysis procedure and supporting methodological references to the Data synthesis section. The corresponding findings were added to the gait variability and gait asymmetry Results sections, and the complete results under each assumed correlation are provided in S3 Table. We also added the following qualification to the Discussion:

“Although the gait-variability findings were robust across the assumed within-study correlations, the gait-asymmetry findings varied according to the assumed dependence among metrics and should therefore be interpreted cautiously.”

Finally, some separately reported metric-level data, particularly those from Micó-Amigo et al., were available only as rounded values. We have acknowledged that this may have reduced the precision of the metric-level sensitivity analysis.

New references:

Borenstein M, Hedges LV, Higgins J, Rothstein HR. Introductionto meta-analysis2009.

Riley RD. Multivariate meta-analysis: the effect of ignoring within-study correlation. J R Stat Soc Ser A Stat Soc. 2009;172(4):789-811. doi: <https://doi.org/10.1111/j.1467-985X.2008.00593.x>.

3. The multi-IMU variability result is statistically significant yet the 95% prediction interval still includes the null—how do the authors interpret the clinical utility of this finding for individual-level discrimination?

**Response:**

Thank you for highlighting this important distinction. The statistically significant pooled effect for the multi-IMU subgroup (Hedges’ g = 0.397, 95% CI 0.221 to 0.574; *p* < 0.001) indicates that, on average, individuals with PD demonstrated greater gait variability than healthy controls across the three included studies. However, the 95% prediction interval was wide and included the null (-0.746 to 1.540), indicating that a future comparable study could observe little or no group-level difference. The small number of studies contributing to this subgroup further limits the precision and generalizability of the pooled estimate.

We also clarify that neither the pooled standardized mean difference nor its prediction interval directly evaluates individual-level discrimination. Hedges’ g quantifies the average separation between groups, but the distributions of gait variability in individuals with PD and healthy controls may still overlap substantially. Establishing clinical utility at the individual level would require diagnostic-accuracy analyses reporting measures such as the area under the receiver-operating-characteristic curve, sensitivity, specificity, and validated classification thresholds, preferably with external validation. These outcomes could not be synthesized from the available studies.

Furthermore, the difference between the one-IMU and multi-IMU subgroups was not statistically significant (Q between = 0.08, *p* = 0.783). Therefore, the present analysis does not demonstrate that multi-IMU systems provide superior individual-level discrimination compared with single-IMU systems. We have removed or moderated statements concerning “practical importance” and now interpret the multi-IMU result as preliminary evidence of an average group-level difference rather than evidence of established clinical or diagnostic utility.

**The Discussion has been revised to state:**

“Studies using more than one IMU showed significantly greater gait variability in individuals with PD than in HC, whereas studies using one IMU showed no statistically significant difference. However, the difference between these subgroups was not statistically significant, and both prediction intervals included null value. Therefore, the findings do not demonstrate that increasing the number of IMUs improves diagnostic performance. The statistically significant pooled multi-IMU effect represents an average group-level difference and does not directly establish individual-level discrimination. Because only three studies contributed to this subgroup and its 95% prediction interval included the null, its clinical utility remains uncertain [35, 52, 53]. In the primary analysis, no significant gait asymmetry difference was observed in either IMU-number subgroup. Although the gait variability findings were robust across the assumed within-study correlations, the gait asymmetry findings varied according to the assumed dependence among metrics and should therefore be interpreted cautiously.”

“Third, given that the studies used different outcomes related to gait variability (e.g., step time variability, stance time variability, swing time variability, step length variability, step velocity variability, etc.) or asymmetry (e.g., step time asymmetry, stance time asymmetry, swing time asymmetry, step length asymmetry, etc.), these outcomes were combined within studies before Hedges’ g was calculated. This approach did not explicitly account for correlations among multiple outcomes measured in the same participants and may have affected both the magnitude and precision of the pooled estimates. However, sensitivity analyses that treated the metrics separately showed that the gait variability findings were robust across assumed correlations, whereas the gait asymmetry findings varied according to these assumptions. Future studies should report individual gait parameters together with their covariance or correlation matrices, allowing multivariate analyses that appropriately account for within-study dependence.”

“While we must admit that only three studies contributed to this subgroup, its 95% prediction interval included the null, and the difference between the one-IMU and multi-IMU subgroups was not statistically significant [35, 52, 53]. This finding therefore represents preliminary evidence of an average group-level difference rather than evidence of individual-level discrimination or superior clinical utility for multi-IMU systems.”

**The Conclusion has also been revised to state:**

“Gait variability measured by using more than one IMU was significantly greater in individuals with PD than in HC. However, the small number of studies, the null-inclusive prediction interval, and the nonsignificant between-subgroup comparison preclude conclusions about diagnostic utility. Therefore, a need exists for additional, well-designed, cross-sectional trails on this topic.”

4. Why were body-position subgroups (lower-back vs. other) abandoned solely on sample size, and was any exploratory analysis performed?

**Response:**

Thank you for requesting clarification. The body-position analysis was not omitted solely because of a small overall sample. Rather, the distribution of sensor placements did not provide two meaningful subgroups for comparison. Among the seven studies included in the gait-variability analysis, six used at least one sensor on the lower back, lumbar region, or pelvis. Only Warlop et al. did not use a lower-back sensor and instead placed the sensor at the lateral malleolus. Among the seven gait asymmetry studies, all used at least one sensor on the lower back, lumbar region, or pelvis. Consequently, the “other-position” subgroup would have contained only one study for gait variability and no studies for gait asymmetry.

We have revised the manuscript to clarify that the placement subgroup analysis was not performed because of the absence of an adequate comparison group and the confounding between sensor placement and system configuration, rather than simply because of the total sample size.

**The Discussion now states:**

“A formal comparison of lower back versus other sensor placements was not possible because only one gait variability study did not include a lower back, lumbar, or pelvic sensor [51], and no corresponding comparator study was available for gait asymmetry. Moreover, several multi-IMU systems included sensors at both lower back and other body positions, preventing the effects of placement from being separated from sensor number and system configuration.”

5. Several included studies mixed straight walking and turning; was turning-related variability examined separately, and if not, why?

**Response:**

Thank you for raising this important point. We did not conduct a separate meta-analysis of turning-related variability. Although several protocols included a change of direction, only Meng et al. reported variability metrics separately for straight walking and 180° turning. Micó-Amigo et al. evaluated continuous circular walking, which was not considered directly comparable with a discrete turning phase. The remaining studies either did not report turn-specific summary data separately or calculated their gait outcomes across the overall walking protocol. Consequently, there was insufficient comparable data to estimate a pooled turning-specific effect.

We agree that combining evidence obtained from straight walking, discrete turning, and continuous circular walking may introduce protocol-related heterogeneity and may obscure gait disturbances that are particularly apparent during turning. We have therefore acknowledged this issue in the Strengths and Potential Limitations section and recommended that future studies report these phases separately using standardized definitions and protocols.

**The revised text states:**

“Fifth, sensor placement could not be examined independently because nearly all studies included a lower-back, lumbar, or pelvic sensor. Multi-IMU systems also frequently combined these sensors with other placements. Consequently, placement was confounded with sensor number and system configuration, and an adequate comparator subgroup was unavailable.”
