# Supplementary material for: The ability of the commercial inertial measurement unit-based gait system in determining Parkinson’s disease: A systematic review with meta-analysis of cross-sectional studies
Source: PLoS One. 2026 Oct 15;21(10):e0360793. doi: 10.1371/journal.pone.0360793 (PMC13671562; doi:10.1371/journal.pone.0360793)
Supplement: Peer review history

# Peer review: Decision Letter 0

**DOI:** 10.1371/journal.pone.0360793.r001

**Authors:** Tadashi Ito

30 Jul 2026

Dear Dr. Tang,

Thank you for submitting your manuscript to PLOS ONE. After careful consideration, we feel that it has merit but does not fully meet PLOS ONE’s publication criteria as it currently stands. Therefore, we invite you to submit a revised version of the manuscript that addresses the points raised during the review process.

Please submit your revised manuscript by Sep 13 2026 11:59PM. If you will need more time than this to complete your revisions, please reply to this message or contact the journal office at plosone@plos.org. When you're ready to submit your revision, log on to https://www.editorialmanager.com/pone/ and select the 'Submissions Needing Revision' folder to locate your manuscript file.

- A letter that responds to each point raised by the academic editor and reviewer(s). You should upload this letter as a separate file labeled 'Response to Reviewers'.
- A marked-up copy of your manuscript that highlights changes made to the original version. You should upload this as a separate file labeled 'Revised Manuscript with Track Changes'.
- An unmarked version of your revised paper without tracked changes. You should upload this as a separate file labeled 'Manuscript'.

If you would like to make changes to your financial disclosure, please include your updated statement in your cover letter. Guidelines for resubmitting your figure files are available below the reviewer comments at the end of this letter.

If applicable, we recommend that you deposit your laboratory protocols in protocols.io to enhance the reproducibility of your results. Protocols.io assigns your protocol its own identifier (DOI) so that it can be cited independently in the future. For instructions see: https://journals.plos.org/plosone/s/submission-guidelines#loc-laboratory-protocols. Additionally, PLOS ONE offers an option for publishing peer-reviewed Lab Protocol articles, which describe protocols hosted on protocols.io. Read more information on sharing protocols at https://plos.org/protocols?utm_medium=editorial-email&utm_source=authorletters&utm_campaign=protocols.

As the corresponding author, your ORCID iD is verified in the submission system and will appear in the published article. PLOS supports the use of ORCID, and we encourage all coauthors to register for an ORCID iD and use it as well. Please encourage your coauthors to verify their ORCID iD within the submission system before final acceptance, as unverified ORCID iDs will not appear in the published article. *Only*  the individual author can complete the verification step; PLOS staff *cannot*  verify ORCID iDs on behalf of authors.

We look forward to receiving your revised manuscript.

Kind regards,

Tadashi Ito

Academic Editor

PLOS One

Journal Requirements:

When submitting your revision, we need you to address these additional requirements.

1. Please ensure that your manuscript meets PLOS ONE's style requirements, including those for file naming. The PLOS ONE style templates can be found at

https://journals.plos.org/plosone/s/file?id=wjVg/PLOSOne_formatting_sample_main_body.pdf and

https://journals.plos.org/plosone/s/file?id=ba62/PLOSOne_formatting_sample_title_authors_affiliations.pdf

2. Thank you for stating the following in your Competing Interests section:

“No authors have competing interests”

Please complete your Competing Interests on the online submission form to state any Competing Interests. If you have no competing interests, please state "The authors have declared that no competing interests exist.", as detailed online in our guide for authors at http://journals.plos.org/plosone/s/submit-now

This information should be included in your cover letter; we will change the online submission form on your behalf.

3. When completing the data availability statement of the submission form, you indicated that you will make your data available on acceptance. We strongly recommend all authors decide on a data sharing plan before acceptance, as the process can be lengthy and hold up publication timelines. Please note that, though access restrictions are acceptable now, your entire data will need to be made freely accessible if your manuscript is accepted for publication. This policy applies to all data except where public deposition would breach compliance with the protocol approved by your research ethics board. If you are unable to adhere to our open data policy, please kindly revise your statement to explain your reasoning and we will seek the editor's input on an exemption. Please be assured that, once you have provided your new statement, the assessment of your exemption will not hold up the peer review process.

4. Please include captions for your Supporting Information files at the end of your manuscript, and update any in-text citations to match accordingly. Please see our Supporting Information guidelines for more information: http://journals.plos.org/plosone/s/supporting-information.

5. If the reviewer comments include a recommendation to cite specific previously published works, please review and evaluate these publications to determine whether they are relevant and should be cited. There is no requirement to cite these works unless the editor has indicated otherwise.

[Note: HTML markup is below. Please do not edit.]

Reviewers' comments:

Reviewer's Responses to Questions

**Comments to the Author**

1. Is the manuscript technically sound, and do the data support the conclusions?

Reviewer #1: Yes

**********

2. Has the statistical analysis been performed appropriately and rigorously?

Reviewer #1: Yes

**********

3. Have the authors made all data underlying the findings in their manuscript fully available?

The PLOS Data policy

Reviewer #1: Yes

**********

4. Is the manuscript presented in an intelligible fashion and written in standard English?

Reviewer #1: Yes

**********

Reviewer #1: Thank you for the opportunity to review this interesting manuscript examining the ability of IMU-based gait systems to distinguish individuals with Parkinson’s disease (PD) from healthy controls. The topic is clinically relevant, and the systematic approach taken by the authors is valuable. I would like to offer several comments that may help strengthen the manuscript.

1.Consistency between the Introduction and the study objective

The Introduction emphasizes the importance of monitoring the progression of motor symptoms in PD and the associated economic burden. However, the primary aim of the present study is to evaluate the ability of IMU-based gait measures to distinguish individuals with PD from healthy controls. To improve the overall coherence of the manuscript, the Introduction may benefit from placing greater emphasis on the clinical significance of early detection and differential identification between PD and healthy individuals, rather than disease progression.

2.Clinical interpretation of gait variability

The manuscript reports that gait variability measured by more than one IMU sensor is associated with distinguishing PD from healthy controls. However, the clinical interpretation of “gait variability” remains somewhat unclear. From a clinical perspective, it would be helpful if the authors could discuss what aspects of PD gait this variability might reflect—for example, whether it is more closely related to freezing of gait, general shuffling gait, or other gait disturbances commonly observed in PD. Adding such discussion would enhance the clinical relevance of the findings.

3.Lack of detectable gait asymmetry

Parkinson’s disease is typically characterized by motor asymmetry, particularly in the early stages. Therefore, the absence of a significant difference in gait asymmetry between PD and healthy controls may appear somewhat unexpected from a clinical standpoint. It would be helpful if the authors could discuss possible explanations for this finding. For instance, the placement of sensors (e.g., on the trunk or lower limbs) may limit the ability to detect certain asymmetric features such as reduced arm swing.

4.Consideration of cost-effectiveness

Since the Introduction discusses the economic burden of PD and presents IMU systems as a potentially low-cost solution, it would be useful to include some discussion of the practical cost-effectiveness of the system. For example, how does the cost or practicality of this approach compare with other commonly used clinical assessments or diagnostic methods? Even a brief discussion would strengthen the practical implications of the study.

5.Other parkinsonian disorders

The present analysis focuses on distinguishing PD from healthy controls. However, in clinical practice, an important diagnostic challenge is the differentiation of PD from other parkinsonian disorders, such as multiple system atrophy (MSA) or progressive supranuclear palsy (PSP). Since these conditions were not considered in the analysis, it would be appropriate to acknowledge this point as a limitation of the study.

6.Transparency of the consensus process

In the Methods section, the manuscript states that “disagreements were resolved by consensus.” While this is common practice in systematic reviews, the description would benefit from slightly greater transparency. If possible, the authors could briefly clarify what types of disagreements occurred and how consensus was achieved.

Overall, this study addresses an important topic, and with some clarification and expansion of the discussion, the manuscript could provide valuable insights into the potential role of IMU-based gait analysis in PD research.

**********

what does this mean?). If published, this will include your full peer review and any attached files.

If you choose “no”, your identity will remain anonymous but your review may still be made public.

**Do you want your identity to be public for this peer review?**  For information about this choice, including consent withdrawal, please see our Privacy Policy

Reviewer #1: No

**********

[NOTE: If reviewer comments were submitted as an attachment file, they will be attached to this email and accessible via the submission site. Please log into your account, locate the manuscript record, and check for the action link "View Attachments". If this link does not appear, there are no attachment files.]

To ensure your figures meet our technical requirements, please review our figure guidelines: https://journals.plos.org/plosone/s/figures

You may also use PLOS’s free figure tool, NAAS, to help you prepare publication quality figures: https://journals.plos.org/plosone/s/figures#loc-tools-for-figure-preparation.

NAAS will assess whether your figures meet our technical requirements by comparing each figure against our figure specifications.

# Author comment: Author response to Decision Letter 1

**DOI:** 10.1371/journal.pone.0360793.r002

5 Aug 2026

We would like to appreciate the opportunity to revise and resubmit our manuscript. We have seriously considered all the comments and carefully revised the manuscript accordingly. Revisions are highlighted in the manuscript using a red color to indicate where changes have taken place. We feel that the quality of the manuscript has been significantly improved with these modifications and improvements based on the reviewers’ suggestions and comments.

Attachment Submitted filename: Response to reviewers.docx

# Peer review: Decision Letter 1

**DOI:** 10.1371/journal.pone.0360793.r003

**Authors:** Gary Guerra

20 Sep 2026

Dear Dr. TANG,

Thank you for submitting your manuscript to PLOS One. After careful consideration, we feel that it has merit but does not fully meet PLOS One’s publication criteria as it currently stands. Therefore, we invite you to submit a revised version of the manuscript that addresses the points raised during the review process.

Please submit your revised manuscript by Nov 04 2026 11:59PM. If you will need more time than this to complete your revisions, please reply to this message or contact the journal office at plosone@plos.org. When you're ready to submit your revision, log on to https://www.editorialmanager.com/pone/ and select the 'Submissions Needing Revision' folder to locate your manuscript file.

- A letter that responds to each point raised by the academic editor and reviewer(s). You should upload this letter as a separate file labeled 'Response to Reviewers'.
- A marked-up copy of your manuscript that highlights changes made to the original version. You should upload this as a separate file labeled 'Revised Manuscript with Track Changes'.
- An unmarked version of your revised paper without tracked changes. You should upload this as a separate file labeled 'Manuscript'.

If you would like to make changes to your financial disclosure, please include your updated statement in your cover letter. Guidelines for resubmitting your figure files are available below the reviewer comments at the end of this letter.

If applicable, we recommend that you deposit your laboratory protocols in protocols.io to enhance the reproducibility of your results. Protocols.io assigns your protocol its own identifier (DOI) so that it can be cited independently in the future. For instructions see: https://journals.plos.org/plosone/s/submission-guidelines#loc-laboratory-protocols. Additionally, PLOS One offers an option for publishing peer-reviewed Lab Protocol articles, which describe protocols hosted on protocols.io. Read more information on sharing protocols at https://plos.org/protocols?utm_medium=editorial-email&utm_source=authorletters&utm_campaign=protocols.

As the corresponding author, your ORCID iD is verified in the submission system and will appear in the published article. PLOS supports the use of ORCID, and we encourage all coauthors to register for an ORCID iD and use it as well. Please encourage your coauthors to verify their ORCID iD within the submission system before final acceptance, as unverified ORCID iDs will not appear in the published article. *Only*  the individual author can complete the verification step; PLOS staff *cannot*  verify ORCID iDs on behalf of authors.

We look forward to receiving your revised manuscript.

Kind regards,

Gary Guerra, Ph.D.

Academic Editor

PLOS One

Journal Requirements:

1. If the reviewer comments include a recommendation to cite specific previously published works, please review and evaluate these publications to determine whether they are relevant and should be cited. There is no requirement to cite these works unless the editor has indicated otherwise.

2. Please review your reference list to ensure that it is complete and correct. If you have cited papers that have been retracted, please include the rationale for doing so in the manuscript text, or remove these references and replace them with relevant current references. Any changes to the reference list should be mentioned in the rebuttal letter that accompanies your revised manuscript. If you need to cite a retracted article, indicate the article’s retracted status in the References list and also include a citation and full reference for the retraction notice.

[Note: HTML markup is below. Please do not edit.]

Reviewers' comments:

Reviewer's Responses to Questions

**Comments to the Author**

Reviewer #1: All comments have been addressed

Reviewer #2: All comments have been addressed

**********

2. Is the manuscript technically sound, and do the data support the conclusions?

Reviewer #1: Yes

Reviewer #2: Yes

**********

3. Has the statistical analysis been performed appropriately and rigorously?

Reviewer #1: Yes

Reviewer #2: Yes

**********

4. Have the authors made all data underlying the findings in their manuscript fully available?

The PLOS Data policy

Reviewer #1: Yes

Reviewer #2: Yes

**********

5. Is the manuscript presented in an intelligible fashion and written in standard English?

Reviewer #1: Yes

Reviewer #2: Yes

**********

Reviewer #1: The authors have adequately addressed all of my previous comments and have revised the manuscript accordingly.

I have no further comments and recommend acceptance for publication.

Reviewer #2: *Critique*

This is a timely and well-structured PRISMA-guided meta-analysis on a clinically relevant question. Strengths include dual independent screening/extraction, use of the IVhet model, Doi plots/LFK for small-study effects, clear subgrouping by number of sensors, and transparent reporting of heterogeneity and prediction intervals. The main finding—that multi-IMU gait variability (but not asymmetry or single-IMU measures) shows a statistically significant difference—is cautious and appropriately tempered by the small number of studies and residual confounding risk.

Key limitations that weaken the strength of the conclusions:

- Very small evidence base (only 7–9 studies; subgroups even smaller), so the “practically important” claim for multi-IMU variability rests on limited data and is vulnerable to single-study influence.

- Substantial clinical and methodological heterogeneity (different commercial systems, sensor placements, walking protocols, PD stages, medication status, and pooling of multiple variability/asymmetry metrics via decomposition) is only partially addressed; the non-overlapping CIs criterion for subgroup differences is conservative but under-powered.

- Risk-of-bias assessment correctly flags frequent confounding and sampling issues, yet these are not quantitatively explored (no meta-regression).

- Pooling heterogeneous spatiotemporal metrics into single “variability” or “asymmetry” outcomes risks under-estimating true effects and obscures which specific parameters drive discrimination.

- Generalizability to early/prodromal PD and differential diagnosis versus other parkinsonisms remains untested, limiting translational impact.

Overall, the paper is methodologically sound for its scope but over-reaches slightly in language about practical importance given the sparse, heterogeneous data.

*Questions*

1. Why was the primary analysis performed on mean differences rather than standardized mean differences throughout, and how sensitive are the multi-IMU findings to the choice of effect-size metric?

2. Given that multiple variability (or asymmetry) outcomes were pooled within studies via decomposition of means/SDs, can you provide the within-study correlation assumptions used and any sensitivity analyses that treat the metrics as separate?

3. The multi-IMU variability result is statistically significant yet the 95% prediction interval still includes the null—how do the authors interpret the clinical utility of this finding for individual-level discrimination?

4. Why were body-position subgroups (lower-back vs. other) abandoned solely on sample size, and was any exploratory analysis performed?

5. Several included studies mixed straight walking and turning; was turning-related variability examined separately, and if not, why?

**********

what does this mean?). If published, this will include your full peer review and any attached files.

If you choose “no”, your identity will remain anonymous but your review may still be made public.

**Do you want your identity to be public for this peer review?**  For information about this choice, including consent withdrawal, please see our Privacy Policy

Reviewer #1: No

Reviewer #2: No

**********

[NOTE: If reviewer comments were submitted as an attachment file, they will be attached to this email and accessible via the submission site. Please log into your account, locate the manuscript record, and check for the action link "View Attachments". If this link does not appear, there are no attachment files.]

To ensure your figures meet our technical requirements, please review our figure guidelines: https://journals.plos.org/plosone/s/figures

You may also use PLOS’s free figure tool, NAAS, to help you prepare publication quality figures: https://journals.plos.org/plosone/s/figures#loc-tools-for-figure-preparation.

NAAS will assess whether your figures meet our technical requirements by comparing each figure against our figure specifications.

# Author comment: Author response to Decision Letter 2

**DOI:** 10.1371/journal.pone.0360793.r004

30 Sep 2026

We have revised the manuscript and improved the quality of the current submission.

Thanks!

Attachment Submitted filename: Response_to_reviewers_auresp_2.docx

# Editor report: Decision Letter 2

**DOI:** 10.1371/journal.pone.0360793.r005

**Authors:** Gary Guerra

2 Oct 2026

The Ability of the Commercial Inertial Measurement Unit-based Gait System in Determining Parkinson’s Disease: A Systematic Review with Meta-Analysis of Cross-Sectional Studies

PONE-D-25-51016R2

Dear Dr. Tang,

We’re pleased to inform you that your manuscript has been judged scientifically suitable for publication and will be formally accepted for publication once it meets all outstanding technical requirements.

Within one week, you’ll receive an e-mail detailing the required amendments. When these have been addressed, you’ll receive a formal acceptance letter and your manuscript will be scheduled for publication.

An invoice will be generated when your article is formally accepted. Please note, if your institution has a publishing partnership with PLOS and your article meets the relevant criteria, all or part of your publication costs will be covered. Please make sure your user information is up-to-date by logging into Editorial Manager at Editorial Manager® and clicking the ‘Update My Information' link at the top of the page. For questions related to billing, please contact billing support.

If your institution or institutions have a press office, please notify them about your upcoming paper to help maximize its impact. If they’ll be preparing press materials, please inform our press team as soon as possible -- no later than 48 hours after receiving the formal acceptance. Your manuscript will remain under strict press embargo until 2 pm Eastern Time on the date of publication. For more information, please contact onepress@plos.org.

Kind regards,

Gary Guerra, Ph.D.

Academic Editor

PLOS One

Additional Editor Comments (optional):

Reviewers' comments:

# Editor report: Acceptance letter

**DOI:** 10.1371/journal.pone.0360793.r006

**Authors:** Gary Guerra

PONE-D-25-51016R2

PLOS One

Dear Dr. TANG,

I'm pleased to inform you that your manuscript has been deemed suitable for publication in PLOS One. Congratulations! Your manuscript is now being handed over to our production team.

At this stage, our production department will prepare your paper for publication. This includes ensuring the following:

* All references, tables, and figures are properly cited

* All relevant supporting information is included in the manuscript submission,

* There are no issues that prevent the paper from being properly typeset

You will receive further instructions from the production team, including instructions on how to review your proof when it is ready. Please keep in mind that we are working through a large volume of accepted articles, so please give us a few days to review your paper and let you know the next and final steps.

Lastly, if your institution or institutions have a press office, please let them know about your upcoming paper now to help maximize its impact. If they'll be preparing press materials, please inform our press team within the next 48 hours. Your manuscript will remain under strict press embargo until 2 pm Eastern Time on the date of publication. For more information, please contact onepress@plos.org.

You will receive an invoice from PLOS for your publication fee after your manuscript has reached the completed accept phase. If you receive an email requesting payment before acceptance or for any other service, this may be a phishing scheme. Learn how to identify phishing emails and protect your accounts at https://explore.plos.org/phishing.

If we can help with anything else, please email us at customercare@plos.org.

Thank you for submitting your work to PLOS One and supporting open access.

Kind regards,

PLOS One Editorial Office Staff

on behalf of

Dr. Gary Guerra

Academic Editor

PLOS One
